# Comprehensive review on ultrasound-responsive theranostic nanomaterials: mechanisms, structures and medical applications

**DOI:** 10.3762/bjnano.12.64

**Published:** 2021-08-11

**Authors:** Sepand Tehrani Fateh, Lida Moradi, Elmira Kohan, Michael R Hamblin, Amin Shiralizadeh Dezfuli

**Affiliations:** 1 School of Medicine, Shahid Beheshti University of Medical Sciences, Tehran, Iran; 2 Department of Tissue Engineering and Applied Cell Sciences, School of Advanced Technologies in Medicine, Tehran University of Medical Sciences, Tehran, Iran; 3 Department of Science, University of Kurdistan, Kurdistan, Sanandaj, Iran; 4 Laser Research Centre, Faculty of Health Science, University of Johannesburg, Doornfontein 2028, South Africa; 5 Physiology Research Center, Faculty of medicine, Iran University of Medical Sciences, Tehran, Iran

**Keywords:** smart nanomaterials, sonodynamic therapy, sonoporation, theranostics, ultrasound, ultrasound responsive nanomaterials

## Abstract

The field of theranostics has been rapidly growing in recent years and nanotechnology has played a major role in this growth. Nanomaterials can be constructed to respond to a variety of different stimuli which can be internal (enzyme activity, redox potential, pH changes, temperature changes) or external (light, heat, magnetic fields, ultrasound). Theranostic nanomaterials can respond by producing an imaging signal and/or a therapeutic effect, which frequently involves cell death. Since ultrasound (US) is already well established as a clinical imaging modality, it is attractive to combine it with rationally designed nanoparticles for theranostics. The mechanisms of US interactions include cavitation microbubbles (MBs), acoustic droplet vaporization, acoustic radiation force, localized thermal effects, reactive oxygen species generation, sonoluminescence, and sonoporation. These effects can result in the release of encapsulated drugs or genes at the site of interest as well as cell death and considerable image enhancement. The present review discusses US-responsive theranostic nanomaterials under the following categories: MBs, micelles, liposomes (conventional and echogenic), niosomes, nanoemulsions, polymeric nanoparticles, chitosan nanocapsules, dendrimers, hydrogels, nanogels, gold nanoparticles, titania nanostructures, carbon nanostructures, mesoporous silica nanoparticles, fuel-free nano/micromotors.

## Review

### Introduction

#### Smart drug delivery vehicles

It is well known that the administration of most anticancer drugs can produce considerable systemic toxicity, which in some cases can be dose-limiting. Whether oral administration or intravenous injection is employed, the drug often accumulates in normal healthy tissues and causes damages. Therefore, it is necessary to target and release these drugs at the desired sites in a controlled manner to decrease their systemic side effects and to increase their therapeutic efficiency [1]. To overcome the limitations and drawbacks of conventional drugs, such as uncontrolled release and nonspecific biodistribution, drug delivery systems (DDS) such as liposomes, polymeric nanoparticles, or nanoemulsions (NEs) have been extensively explored. However, even conventional DDS often lack the ability to release the cargo at the desired site in a well-controlled manner. Therefore, smart DDS have been developed to provide drug release at the target site in a spatially and temporally controlled manner, preserve the drug/agent in the target site for a longer time, increase the therapeutic efficacy, and decrease undesirable systemic side effects [2].

Smart DDS (also known as stimulus-responsive drug delivery platforms) can be traced back to the late 1970s when thermosensitive liposomes were introduced. These liposomes could locally release drugs in response to externally applied heat to the tissues [3]. The main goal of stimulus-responsive DDS can be defined as systematic administration combined with local activation. Dual/multi-stimuli-responsive smart delivery systems can be loaded with various bioactive molecules and will only release their cargo in the presence of two or more different stimuli, which can be chemical, biochemical, or physical in nature. These smart/intelligent systems have many advantages and unique potential in drug delivery, tissue engineering, diagnosis, or biological sensors [4]. In order to produce stimulus-responsive platforms, we need to design materials that can undergo specific structural changes, for instance, protonation, cleavage, or conformational changes after exposure to certain stimuli which trigger the release of the cargo [3]. The physicochemical properties of these systems can be changed when triggered by environmental stimuli, such as temperature, pH, enzyme, redox potential, ionic strength, or solvent composition of the media. Other stimuli are external, such as heat, light, electric field, magnetic field, or ultrasound (US) [5–7]. Designing such single, dual, or multi-stimulus-responsive smart delivery vehicles provides an opportunity for the development of new biomaterials. The optimization of their responses to local/environmental stimuli can provide better-controlled drug delivery and superior therapeutic effects through the synergistic effect of various environmental stimuli [8–9]. These systems have been discussed in several review articles [3,7,10–11].

Endogenous or internal stimuli can be hard to control because of the heterogeneous disease environment. On the other hand, the use of exogenous or external stimuli may cause tissue damage and the depth of penetration may not be sufficient to trigger drug release deep inside tissues and organs. However, external triggers may be overall more desirable due to their controllable activation properties [2,10].

Many factors need to be taken into account in the design of smart DDS, such as overcoming biological barriers, selecting the best administration route, minimizing toxicity, ensuring biodegradability, biosafety, and efficacy, and guarding against long-term carcinogenesis [2]. Although animal models cannot accurately simulate every single aspect of human disease, in vivo therapeutic evaluation of these smart nanostructures for drug delivery is important despite the complexity and a large number of parameters to be optimized [2].

However, despite a large amount of innovations and laboratory researches, the efficacy and safety of nanomaterials used as drug carriers should be evaluated through clinical trials in order to be available in the clinical setting [2–3,11–13]. Currently, many studies are in the process of evaluating new applications of stimuli-responsive DDS in different diseases, which have been covered in some review articles [1–2,14–18].

#### General concept of ultrasound-responsive cargo delivery

One of the most significant advantages of stimuli-responsive DDS is the precise spatial and temporal control of drug release in response to the application of exogenous or endogenous stimuli, including US [3]. Ultrasound is traditionally used in diagnostic medicine but now it is finding a place in drug delivery in combination with specific nanoparticles (NPs). The use of US in drug delivery has expanded greatly since the first report in 1989 [19]. Nowadays, advances made in new US-sensitive smart carriers have led US to become an effective technique to trigger drug delivery at targeted sites by tuning the power density, frequency, time of exposure, duty cycles, and the position of the acoustic transducer [20–21].

There are many different parameters that need to be considered in the design of efficient US-responsive systems. These systems should be stable and be able to properly encapsulate various types of cargo without any leakage before ultrasonication. They should then release the cargo after US stimulation and also, in some cases, have the ability to be monitored via imaging modalities. Many US-responsive NPs have been reported as part of theranostic systems, which can be used for both therapy and imaging at the same time [22–23].

US can activate drug release and delivery through various mechanisms [24]. As the longitudinal pressure wave propagates in a tissue, a fraction of the energy is absorbed by the tissue or by the drug carrier, resulting in local heating [25–26]. Thermosensitive structures can release their cargo in response to locally elevated temperatures [24]. Under some circumstances, small mechanical displacements of the tissue can result in nucleation, growth, and collapse of gas bubbles in a process known as acoustic cavitation, which is responsible for drug release from some structures [27]. In other cases, disruption and destabilization of the complex nanostructure subsequent to US vibration leads to drug release [28–30]. In addition, the ultrasonication of certain complexes can generate free radicals that can cause cell damage or activation of cellular signaling pathways [31]. Early reports in the field of ultrasonic drug delivery demonstrated that the application of US energy alone could facilitate intracellular delivery of molecules by altering membrane integrity or interfering with the endocytosis process; however, this could also be harmful to the cells under some conditions [32–35]. Acoustic impedance, attenuation, acoustic power, intensity, frequency, beam shape, and exposure time are important parameters for the utilization of US devices. Moreover, the anatomical location of the US application and the characteristics of the transduction medium should be considered [30,36].

Many different smart or stimulus-responsive drug carriers have been used in combination with US. These include exosomes, liposomes, polymeric, organic or inorganic hybrid NPs, as well as other nanomaterials to control drug release behavior as well as to investigate their potential clinical applications, which have been discussed in several papers [23,37–42].

US-enhanced drug delivery has several important advantages since it is noninvasive, can be precisely focused and controlled, and can penetrate deep into the body [24]. In addition, ultrasonic waves have some unique characteristics as an extracorporeal tool to increase drug permeability and drug release across biological barriers with the goal of treating human solid cancers [14,18,43], such as kidney [44], prostate [45–47], liver [48], lung [49], cardiovascular [50], breast [51–53], pancreatic [54–55], and brain [56–59] tumors.

#### Physics of ultrasound

Ultrasound is a noninvasive and nonionizing acoustic wave with a frequency above 20 kHz, which is based on the human perception of sound. The range of US frequency used in medical applications varies from 1 to 15 MHz, in which 1 MHz frequency is used for therapeutic applications and 2.5 to 15 MHz for diagnostic procedures according to the depth and type of the organ or tissue and the physics of the mechanical wave propagation [60]. Sound is a back-and-forth mechanical motion or vibration of molecules in a medium that transports energy [60]. Ultrasound is generally produced by the passage of electric current through a piezoelectric crystal [61].

The interaction of acoustic waves with the interfaces that exist between different tissues causes an alteration in the energy of the US. When these waves encounter tissues with different values of acoustic impedance (a parameter that mainly depends on the tissue density), a proportion of the wave energy is reflected while the remainder passes through the tissue in a process called transmission. Other consequences are the refraction and diffraction of the acoustic wave. Also, a proportion of the energy will be absorbed by the tissues which leads to an increase in temperature. Therefore, this wave gradually loses energy due to absorption, reflection, diffraction, and refraction, which is called attenuation [61]. In solid materials, US may propagate as both longitudinal and transverse waves; however, in fluids and in the majority of soft tissues, the propagation is primarily longitudinal [62].

#### Scope of this review

The main goal of this review is to present a rational design paradigm for the creation of US-responsive theranostic systems which scientists and engineers can use in their quest for more potent treatment and diagnostic procedures. In this review, the mechanisms of action of US-responsive nanomaterials, including cavitation, acoustic radiation force (ARF), phase transition, reactive oxygen species (ROS) production, and hyperthermia will be discussed in the first step. A distinguishable feature of this review is a comprehensive explanation of the mechanisms of action of US-responsive nanomaterials which would help researchers to understand the fundamentals of this field to design and create novel US-responsive nanomaterials. In addition to reviewing the recent literature on this subject, understanding how US affects tissues and nanomaterials might also lead to the introduction of other nonconventional nanomaterials to this field. We then discuss the rational design of some state-of-the-art materials for US-triggered drug delivery and review recent progress of each type of drug carrier. The imaging applications of these materials will also be discussed. These materials include nanocarrier formulations and nanostructured contrast agents, such as microbubbles (MBs), surfactant-based carriers (including micelles, NEs, and niosomes), polymer-based carriers (including gels, dendrimers, and capsules), lipid-based carriers (including liposomes and solid lipid NP), and non-polymer-based structures (including nanomachines, gold NPs, titanium, carbon, and silica nanostructures) along with some other novel NPs which can trigger drug release after US activation. A discussion on these less-discussed US-responsive nanomaterials in addition to the conventional nanomaterials (i.e., microbubbles, micelles, liposomes, and nanoemulsions) is another distinguishable feature of this review. Ultrasound-responsive nanomaterials are discussed in terms of their background, structure, preparation methods, advantages and disadvantages, mechanism of action, and recent relevant researches.

**Figure 1:**
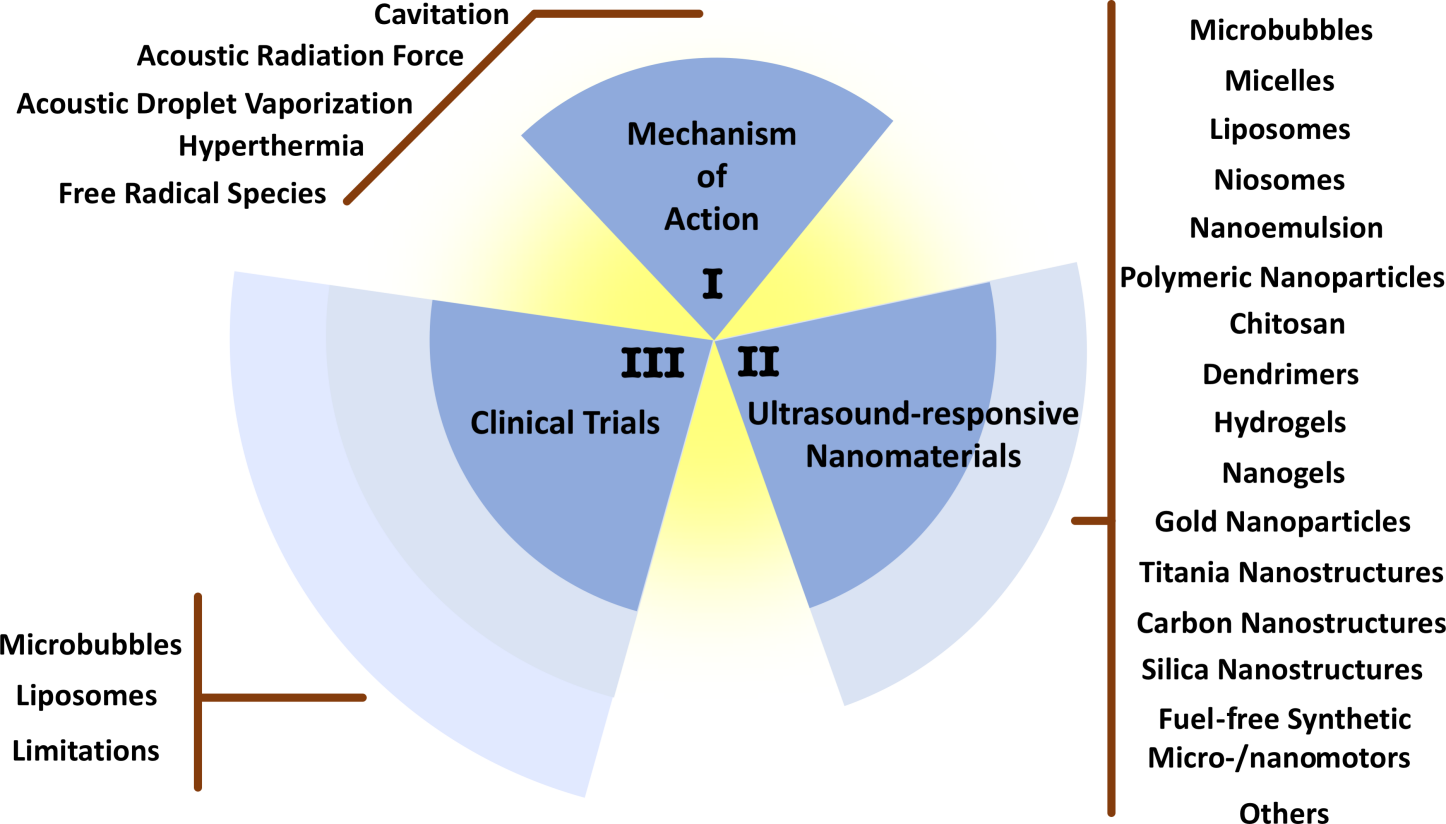
Schematic illustration of the contents.

Finally, the clinical trials on US-responsive nanomaterials are presented and discussed. A summary on the content of this review can be found in Figure 1.

### Mechanisms of action of ultrasound-induced drug release

**Figure 2:**
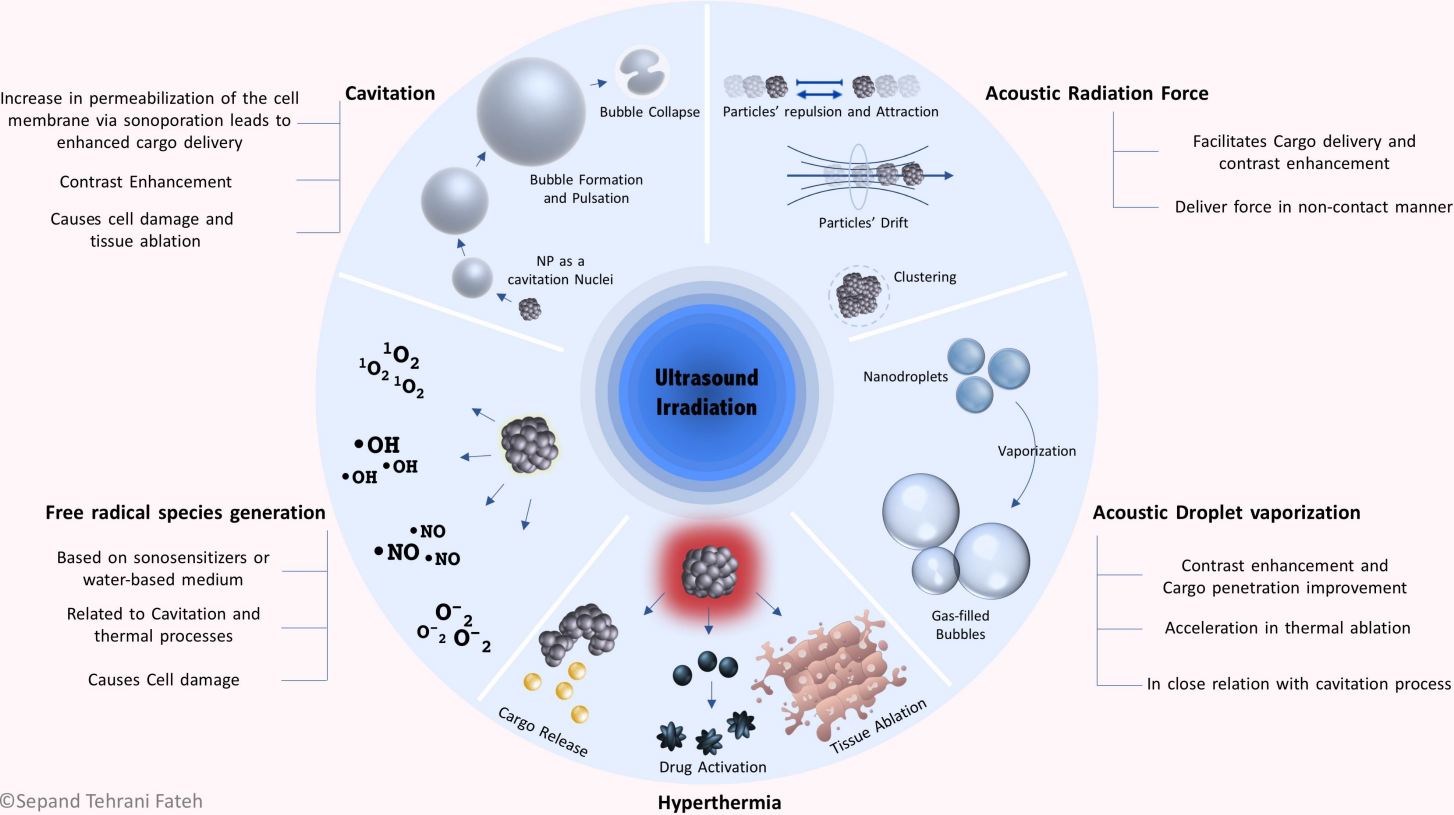
Mechanism of US in synergism with nanomaterials. Generally, the effects of US can be explained by five mechanisms, including cavitation, ARF, acoustic droplet vaporization (ADV), hyperthermia, and free radical species generation. In some conditions, these mechanisms occur at the same time and cannot be studied separately. In this sense, free radical generation is related to cavitation and thermal processes and ADV is integrated with cavitation. As a result of these mechanisms, cargo carriers can release their contents and some drugs become activated. Free radicals cause intrinsic tissue damage, tissue ablation occurs, particles pass through barriers and accumulate in the desired location, and image contrast is enhanced due to the increased backscattered signal.

The application of US would affect the tissues and US-responsive nanomaterials through five distinct mechanisms, leading to the therapeutic or diagnostic activities of US-responsive nanomaterials. Cavitation, acoustic radiation force, acoustic droplet vaporization, hyperthermia, and free radical species generation are recognized as the mechanism of action of US-responsive nanomaterials. These nanomaterials can act through at least one of the mechanisms. Cargo release, drug activation, cell damage, and enhanced cargo penetration, in addition to contrast enhancement, are the clinically practical consequences of the mentioned mechanisms of action (Figure 2). Each mechanism is comprehensively discussed in the following sections.

#### Cavitation

Cavitation has been defined in multiple ways by different authors. Wu et al. defined cavitation as follows: “acoustic cavitation refers to activities associated with air or gas bubbles, pockets, and cavities under excitation of acoustic waves” [63]. Brennen defined cavitation as “the process of rupturing a liquid by a decrease in pressure at a roughly constant liquid temperature” [64], while Paliwal and Mitragotri stated that cavitation is “the process of formation, pulsation, and collapse of gas-filled cavities in ultrasonicated materials” [27]. Cavitation can be defined as the perturbation of materials by US energy and their interaction with acoustic waves, which leads to a displacement in less dense materials and the subsequent formation of bubbles [27,62]. Cavitation may have both positive and negative impacts on living biological systems, which have been reviewed in [65]. The schematic illustration of this mechanism is presented in Figure 2.

It has been widely reported that US has the potential to trigger intracellular delivery of both low and high molecular weight molecules, such as nucleic acids, proteins, peptides, calcein, dextran [66–70], or gene complexes [67] via a process known as “sonoporation” or “transient cavitation”. Cavitation events are sometimes triggered by the effects of US on MBs. Microbubbles are micrometer-sized (1–10 µm) gas-filled structures that are stabilized by a lipid, surfactant, protein, or polymer shell, whose stiffness or rigidity can affect the final outcome of the MBs upon exposure to US [71–73]. During the cavitation event, backscattered energy leads to the expanding and shrinking of the MBs, which intensifies the biophysical effects of the US waves [41]. This leads to transient permeabilization of cell membranes through the formation of transient pores and/or defects in the lipid bilayer, and finally, the diffusion of surrounding molecules into the cytosol [74].

Cavitation events triggered by MBs reinforce the biophysical effects of using US for drug delivery purposes. Two types of MB cavitation depending on the US intensity have been described: noninertial cavitation and inertial cavitation. Noninertial or stable cavitation events occur at low acoustic pressures [72]. Several in vitro studies have provided evidence that during the expansion phase of MB-triggered cavitation there is a net influx of gas into the MB. The bubbles expand until they reach their resonant size with low amplitude oscillations in a linear direction. These stable oscillations result in the creation of a liquid flow surrounding the MBs, leading to the term “microstream”. Depending on the US intensity, the oscillating MBs come into close proximity with the cells and induce stresses on the cell membrane [75]. Consequently, the triggered shear forces cause disruption of the cell membrane and increase intracellular uptake of drugs which subsequently provides biological effects [76]. The inertial cavitation phenomenon occurs at higher acoustic intensities [74,77] and the MBs oscillate in an asymmetric non-linear manner. This leads to the collapse, implosion, and finally to the fragmentation of the MBs located in close proximity to the cell membrane. It has been shown that during inertial cavitation, in addition to direct oscillating MB–cell membrane interactions, a fluid microjet formed around the MBs can be responsible for providing secondary mechanical stress on the cell membrane and create transient disruption. In fact, microjets can act as a microsyringe for delivering drugs into the cytosol during the collapse phase of the MB cavitation [74].

The maximal distance between the MB and the cell membrane should not exceed one MB diameter in order to exert a significant impact on the cell membrane [78]. Yu et al. reported that when the distance between the cell and the MB was increased to 5.5 µm, the exerted shear stress on the cell membrane suddenly decreased [78].

Schlicher et al. exposed prostate cancer cells (DU145) to 24 kHz US irradiation to investigate the cavitation events and the changes in the cell membrane after sonication. They provided evidence that during US exposure and sonoporation, repairable disruption and an increase in plasma membrane permeability occurred. They also suggested that the change in the cell membrane depended on the intensity of the US waves and the cavitation process. They further suggested that the same results would be obtained if a higher frequency US was employed [79].

Both mechanical stress and chemical effects induced by US could be responsible for the formation of repairable cell membrane pores [74]. Van Wamel et al. reported that the mechanism leading to enhanced cell membrane disruption was a direct interaction between the cell membrane and stable cavitation MBs located close to the cell membrane [80]. The ARF could displace the oscillating MBs several micrometers closer to the cell surface in the direction of the US beam. These cell-targeted MBs generated at a lower US intensity are able to gently pull, compress, and collapse against the cell membrane [81]. These events result in the generation of small MBs, which serve as new cavitation/sonoporation nuclei. The MBs can grow in size and then collapse, eventually leading to the generation of shock waves. These processes can create a pressure of up to 100 atm and increase the local temperature [82]. Taken together, these processes can produce considerable stress on the cells, disrupt the cell membrane, and cause changes in the cell membrane to allow the direct entrance of the cargo into the cell cytoplasm through simple diffusion. Moreover, these stresses can activate cellular stress signaling pathways [83].

Previous studies have concluded that two mechanisms could be involved in US-mediated drug delivery and cell uptake of impermeable molecules: sonoporation and increased endocytosis [84–85]. However, a few studies have demonstrated that the endocytosis pathway is the main mechanism for the delivery of large molecules mediated by US [33,84–85]. Schlicher et al. investigated the uptake and transfer of different molecular weight fluorescent molecules, including calcein, fluorescein isothiocyanate (FITC)-labeled bovine serum albumin (BSA), FITC-labeled 150, 500, and 2000 kDa dextrans into DU145 prostate cancer cells. They blocked the endocytosis mechanism to assess whether the endocytic pathway was upregulated during US exposure. They showed that all of these fluorescent molecules were transferred into the cell cytosol during cavitation, and no major differences were found in the uptake of these molecules. The authors suggested that US may alter the cell membrane integrity, thus enhancing cellular permeability [86]. In another study, De Cock et al. also investigated the uptake of 4 kDa and 2 MDa FITC-dextrans loaded into MBs as model drugs using flow cytometry and FACSCalibur^TM^ (Flow cytometer). They blocked the endocytic pathway and interestingly found two different cell subpopulations after US exposure which had either low or high uptake of FITC-dextran. They found that the “low-uptake” cells showed endocytic uptake, while the ‘high-uptake” cells showed uptake through cell membrane pores [72]. Schoellhammer et al. hypothesized that US could permeabilize the gastrointestinal tract through transient cavitation bubbles. For the first time, they locally demonstrated the intracellular delivery of fluorescently labeled mRNA (≈950 kDa) into the colon of healthy C57BL/6 mice using low-frequency US (40 kHz for 0.5 s). Confocal microscopy showed that the mRNA was safely delivered into the colonic mucosa and the colon tissue of mice, in which the US-mediated delivery of the nucleic acid was administered, had levels of bioluminescence 11-fold higher than the colon tissue of mice that received mRNA alone. This was suggested to be caused by US-induced cavitation, creating transient pores in the plasma membrane which facilitated the cellular diffusion of macromolecules [87]. In another study, the authors assessed the effect of Pluronic P105 micelle-encapsulated doxorubicin (DOX) in the presence of US for the treatment of breast adenocarcinoma tumors in adult female BALB/c mice. The results showed significantly increased accumulation of DOX in the tumor and lower concentrations in distant tissues. They suggested that cavitation bubbles induced by US caused the release of the DOX into the tumor tissue [88]. Similar findings were obtained by Chen et al. They synthesized PC-polyethylene glycol (PEG) liposomes loaded with FITC (FITC-PC-PEG-L) with a diameter ranging from 150 to 200 nm as delivery vehicles in combination with high-intensity focused ultrasound (HIFU) for targeted drug delivery in vivo. The small size of the liposomes allowed for the controlled release of the encapsulated drug. They reported that the application of HIFU (1.1 MHz) for 10 s could release ≈21% of encapsulated FITC from PC-PEG-L liposomes, whereas after sonication for 60 s, the release of FITC was increased to 70%. They suggested that the cavitation events during sonication resulted in rupture and pore-like defects occurring in the cell membrane, leading to the release of DOX and FITC from micelles and liposomes. Thus, the controlled drug release from different carriers in association with US could be employed in clinical settings [89].

Some of the inconsistencies found in the literature reports are likely due to the use of a wide range of US parameters employed in sonoporation studies. The interactions between ultrasonic waves and the surrounding tissue result in different mechanical, chemical, and thermal effects, which in turn lead to different biological effects. It is notable that the studies in favor of the endocytosis pathway often used only modest US intensity. In contrast, the studies claiming that sonoporation is responsible for drug uptake mostly applied higher intensity US [72].

#### Acoustic radiation force

The ARF has been defined as a mechanical force that is generated by the transfer of momentum from the US wave to the medium [60]. The radiation force makes any particles suspended in the fluid drift, form clusters, and attract or repel one another [90]. The acoustic radiation force can be traced back to the publication of Lord Rayleigh in 1902, which was called “the pressure of vibrations” [91]. The radiation force exerted by sound waves was first measured by Altberg [91–92] and the work by King provided the mathematical basis [93].

The ARF can be divided into primary and secondary forces. Primary forces are applied to single particles, while secondary forces cause particle–particle interactions. Moreover, primary forces cause migration and aggregation of the particles in an acoustic field producing nodes and antinodes in steady waves. Secondary forces result in particles approaching closer or moving away from each other [90]. When these forces are applied to gas bubbles, they are called Bjerknes forces, while forces between solid particles are referred to as König forces [90].

King investigated the primary ARF and provided a number of equations to describe this phenomenon. Different parameters are involved in these equations, including fluid density, the complex amplitude of the velocity potential of the imposed sound field, angular driving frequency, speed of sound in the fluid, density of the sphere, and the distance between the center of a sphere and the nearest velocity node plane of the standing sound wave. The theory of King explained this phenomenon and provided some insight about the accumulation of particles in nodes and antinodes of sound fields. Afterward, other investigations evolved these equations to describe other particles with different compositions and also improved the theoretical explanation [90,93].

As mentioned previously, the concept of secondary forces explains the interaction between various particles. Each interaction between these particles can be explained using different equations [90]. An interaction between two bubbles is called Bjerknes. An interaction between a bubble and a solid particle, according to the related equation, implies that particles denser than the host liquid are attracted by the bubble, while particles less dense are repelled by it. This equation also implies that the oscillations of the particle are induced by the scattered field of the bubble alone. Another type of interaction is between a bubble and a liquid droplet. The study of interactions between a gas bubble and a liquid droplet is mainly of biomedical interest. In biomedical ultrasound imaging, one has to deal with the radiation force exerted by pulsating gas bubbles and interactions with the components of blood plasma. Changes in the size of a droplet, the distance between the particles, the density of the drop, and the US frequency all affect the behavior of the interaction force and its properties. The equation that describes an interaction between two rigid spheres is named after Leoing. An interaction between “n” compressible spheres in a compressible fluid is also described in [90].

Although all the previously mentioned equations are applicable under specific conditions, they may have less relevance in other possible conditions. In other words, the previously mentioned studies do not provide a general theory that would be valid for all particle pairs of any conceivable nature, for any separation distance, or for an arbitrary number of particles. The early theory of this phenomenon was based on a large number of simplified assumptions, which restricted its application and did not allow for many experimental observations to be explained. More recently, investigators came up with a new general theory that explained the experimental findings and also predicted some new interesting effects. An approach to such a theory was published by Doinikov in many papers [90,94–100]. According to his studies, the calculation of the ARFs acting in a system of particles of interest can be reduced to the calculation of the linear scattering coefficients of the particles. The details of this theory were reviewed in [90].

The radiation force can be produced by various physical effects, such as changes in the energy density of the propagating waves due to absorption and scattering, spatial variations in energy density in standing acoustic waves, reflection from inclusions, walls or other interfaces, and spatial variations in the propagation velocity [101].

The biomedical significance of the ARF effect was first demonstrated in 1971 by Pond, Woodward, and Dyson, who discovered that red blood cells in the blood vessels in vivo could be collected in standing acoustic waves in a band the size of half a wavelength [101–102]. Microbubbles can be utilized in biomedical applications through synergistic effects they undergo with radiation force effects, both as a contrast agent and as a cargo carrier [103–108]. Many studies have demonstrated further applications of ARF in biomedical fields [109–110]. It is possible to deliver force in a noncontact manner using ARF [90], and ARF has been considered as one possible mechanism in nanostructure-based theranostics [111]. The biomedical applications of ARF have been covered in several reviews by Sarvazyan et al. [91,101,112] and Urban et al. [113]. The schematic illustration of this mechanism is presented in Figure 2.

#### Acoustic droplet vaporization

Acoustic droplet vaporization (ADV) is an US method wherein superheated liquid micron-sized droplets are converted into gas MBs approximately 5–6 times larger in diameter [114]. The pressure needed for converting liquid droplets into gas-filled MBs and also whether the droplets form again depend on parameters such as shape and size of the droplets used and the temperature in the medium [115]. The ADV processes are involved in US contrast imaging and can provide a way to improve the penetration capability of large particles, genes, and cells. They can trigger local drug release and provide better in vivo spatial control by applying mechanical forces of oscillation, expansion, and inertial cavitation from ADV-generated MBs [71,116–118]. The authors of a study showed that ADV resulted in irreversible rather than reversible cavitation. Furthermore, the rate of irreversible cavitation was enhanced with an increase in the concentration of the nanodroplets (NDs), pulse duration, and US amplitude. These findings suggested that cavitation is strongly dependent on the expansion, concentration, and size of the ADV-generated MBs close to the cellular membrane and also the cell–MB distance [119]. Acoustic droplet vaporization shows promise for spatial control and acceleration of the thermal ablation of cancer lesions after vaporization of microdroplets or NDs used as cavitation nuclei during HIFU treatment [120–122]. In addition to being a relatively time-consuming procedure, HIFU has the risk of promoting off-target heating of healthy surrounding tissue. One possible solution is the use of targeted MBs to improve the efficiency of HIFU by decreasing the acoustic energy required to cause heating and lesion formation. Xin et al. used pulsed-wave US and continuous-wave US heating to vaporize perfluoropentane (PFP) droplets for local thermal ablation [123]. They reported that different concentrations of ADV droplets could alter the shapes of the produced MBs from small dots to triangular bubbles, which in turn could affect the volume of the thermal lesion produced. The lesion size was much larger after applying pulsed-wave US combined with continuous-wave US, especially for higher concentrations of PFP droplets. Therefore, a bubble-forming strategy may be useful in the clinical settings because the volume and morphology of the thermal ablation can be controlled by changes in both droplet concentration and acoustic pressure. This approach offers a new opportunity for the optimization of HIFU cancer therapy [123].

Furthermore, ADV-generated MBs can provide contrast-enhanced imaging and increase the temperature during HIFU therapy due to the cavitation produced when using ultrasound contrast agents [115]. ADV-generated MBs with the proper density can facilitate uniform HIFU ablative treatment and enhance therapeutic efficacy by providing local control of energy absorption and minimizing the treatment time and tissue damage [120].

The US-induced vaporization of a NE can also provide the force to increase the penetration and delivery of drug cargos through the skin with the goal of decreasing pain [124–125]. Additionally, these nanosystems offer a more stable solution for sonoporation agents in combination with MBs and they have the capacity for drug conjugation and high specificity for localized vaporization [126]. Nanoemulsions are capable of being converted into MBs after ADV and become subjected to cavitation, thus promoting cellular uptake and delivery of entrapped drug/agents into the desired area [127]. Acoustic droplet vaporization has been carried out with many liquids whose boiling points are close to the body temperature. Fluorocarbons, especially perfluorocarbons (PFCs), are great candidates for ADV because they have low cytotoxicity and low solubility in aqueous media. In the last decade, ADV has been employed for vessel occlusion therapy, drug delivery, HIFU, tissue lesion formation, and molecular imaging [128–129]. Previous studies showed that a combination of two different types of nanosized PFC droplets had the potential to decrease the energy needed to induce ADV and enhance the formation of the HIFU-induced thermal lesions, while MBs alone led to undesirable surface heating and lesion formation [121,130]. By using dual-PFC NDs, the HIFU procedure time could be decreased without enhancing the risk of skin damage [131]. It has been established that ADV-mediated delivery of several chemotherapeutic drugs, including DOX [132], paclitaxel [133], and chlorambucil, could be achieved when loaded into PFC droplets [134]. The NDs could be successfully accumulated in a tumor as a result of ADV [135].

There are two main hypotheses to explain the mechanisms by which US can induce vaporization. One theory proposes that the ultrasonic field interacts with the dispersed medium causing vaporization within the bubble core. The second theory suggests that shock waves from the inertial cavitation, occurring near or within the droplet, cause the dispersed medium to vaporize [135–136].

It has been shown that ADV can decrease cell viability through the disruption of cell membranes [114]. Some researchers have suggested that ADV can cause cell death while increasing the penetration of drugs into endothelial or tumor cells. Therefore, a combination approach using drug-loaded NDs plus US could improve therapeutic efficacy [137]. Yi-Ju Ho et al. showed that vascular disruption induced by NDs plus ADV provided a way to deliver drugs into a hypoxic region of a solid tumor [116]. Ho et al. demonstrated that in addition to cargo release and MB formation, tumor tissue damage also occurred after ADV triggered by US [138]. The schematic illustration of this mechanism is presented in Figure 2.

#### Hyperthermia

Ultrasound has a good ability to penetrate deep within the human body, and its ability to be focused makes it an appropriate source of high energy for clinical therapy in comparison with other external sources of energy [139–140]. After the propagation of ultrasonic waves into the body, both thermal and non-thermal effects have been shown to occur [141]. For instance, when US beams are focused on a targeted tissue, the absorption of acoustic energy by the surrounding fluid or living tissue causes local hyperthermia [83]. In targeted drug delivery strategies, localized heating of the tumor tissue without excessive thermal damage to the surrounding normal tissues is an advantage of using US [140]. Local hyperthermia-induced drug delivery is used for the delivery of drugs to the tumor site triggered by the spatially confined thermal effects of US. This method is aimed at enhancing the therapeutic effect of chemotherapy drugs to avoid side effects due to their undesired distribution into surrounding healthy tissues. This technique has been introduced into the clinical practice as an adjuvant approach for the treatment of various human cancers with satisfactory/acceptable safety and negligible side effects [142]. The US wave produces two biological effects, which are hyperthermia and mechanical effects. These biological effects are commonly due to the transient cavitation phenomenon [143]. Drug delivery systems can, in theory, respond to either thermal or mechanical effects. Drug delivery induced by high-frequency ultrasound is associated with thermal effects, while low-frequency ultrasound is mostly associated with mechanical effects [143]. During hyperthermia, the target tumor tissue is exposed to a high temperature above 47 °C, and thermal ablation occurs by direct destruction of the cancer cells. After sub-ablative local hyperthermia involving a slight increase in the temperature of the target tissue, the permeability of tumor vessels, blood circulation, and interstitial fluid pressure could be improved, and eventually, the level of tumor oxygenation could be elevated [140,144].

Nanoparticles can mediate both thermal and non-thermal interactions of US with human tissue. They have an important role in absorbing the energy delivered by the US waves, increasing the temperature of the target tissue, and subsequently increasing the therapeutic effect of hyperthermia [145]. Accordingly, tumor tissue can be loaded with NPs and then exposed to US waves to provide localized hyperthermia within the tumor while preserving healthy tissue from the undesirable side effects of heating [146]. The local production of heat can trigger drug release, intensify the cytotoxic effect of the loaded drugs, and eventually destroy the tumor cells. Therefore, the overall goal of cancer therapy can be improved by employing a nanotechnology-based hyperthermia approach [147]. The schematic illustration of this mechanism is presented in Figure 2.

#### Free radical species generation

Free radical molecules, such as ROS, NO, HO•, can be generated after the US irradiation interacts with specific components in water-based media, which plays a role in both therapeutic and diagnostic applications [148–149]. Due to the toxicity of free radicals, some chemical compounds called sonosensitizers have been used as sonodynamic therapy agents which produce synergistic effects with US irradiation by generating free radicals [150]. Masuda et al. proposed that there is a relation between the quality and quantity of free radical formation and the frequency of the US applied in the presence of MBs [151]. The combination of free-radical-generating components and other materials could lead to multifunctional complexes with both therapeutic and diagnostic potentials [148,152].

The primary reaction in sonodynamic therapy is the dissociation of water into HO• radicals or the formation of singlet oxygen (^1^O_2_) within the targeted medium. It is thought that US cavitation and thermal effect are the leading causes of ROS production [149,153]. Miyaji et al. investigated the generation of free radicals from water molecules in the presence of US under aerobic conditions. 5.5-Dimethyl-1-pyroline-*N*-oxide was used as a trap for HO• free radicals and analyzed using electron spin resonance microscopy after sonolysis [153]. Various nanostructures have been developed for free radical generation under US irradiation. A novel nanostructure was constructed based on a BNN-type NO-releasing molecule and superparamagnetic iron oxide nanoparticles (SPION)-encapsulated mesoporous silica NPs (MSN) which could generate NO free radicals after US triggering under magnetic resonance imaging (MRI) guidance. According to these studies, US irradiation caused distinct NO release. There was a positive association between increasing the power of the US with the rate of NO generation and the cytotoxic effects of NPs on the cancer cells [148]. Titanium-based NPs have been investigated for sonodynamic therapy [149,154]. You et al. produced hydrophilized titanium dioxide NPs (HTiO_2_) and demonstrated its cytotoxic potential and ROS generation under US treatment. Results showed a 29.7-fold increase in ^1^O_2_ concentration in the treated sample compared to non-treated samples [154]. Other researchers have developed platinum-based NPs for sonodynamic therapy and ROS generation in both extra- and intracellular environments [155]. The schematic illustration of this mechanism is presented in Figure 2.

### Ultrasound-responsive nanomaterials

Various nanomaterials, different in nature, have been applied as US-responsive nanomaterials. The nature of the nanomaterials determines their response to US waves and subsequently defines their further applications. In other words, the mechanism of action of US-responsive nanomaterials mostly depends on their composition. Moreover, their biocompatibility, biodistribution, stability, capacity, and diagnostic efficacy are related to this factor. Lipid- and surfactant-based nanomaterials, polymeric nanomaterials, and metallic and non-metallic nanomaterials, in addition to micro- and nanomotors and some miscellaneous nanomaterials, are discussed in the following sections in terms of their background, structure, preparation methods, advantages and disadvantages, and related recent and prominent researches.

#### Microbubbles

The term microbubble usually refers to a hollow particle filled with a specific gas surrounded by a specific layer that serves as a shell [156]. The beginning of MB development can be traced back to the discovery of a relation between gas bubbles in the bloodstream and the strong US echo detected subsequently to an US irradiation [157]. The main application of MBs was in echocardiography to identify myocardial infarction or coronary artery stenosis [158–159]. It has also been used to assess stroke patients [160], fallopian tube patency [161], and in the detection of ureteric reflux [162]. MB-mediated US effects have also been used as a means of nucleating cavitation in the target tissue to increase the speed and efficacy of medical treatment [163–164].

**Figure 3:**
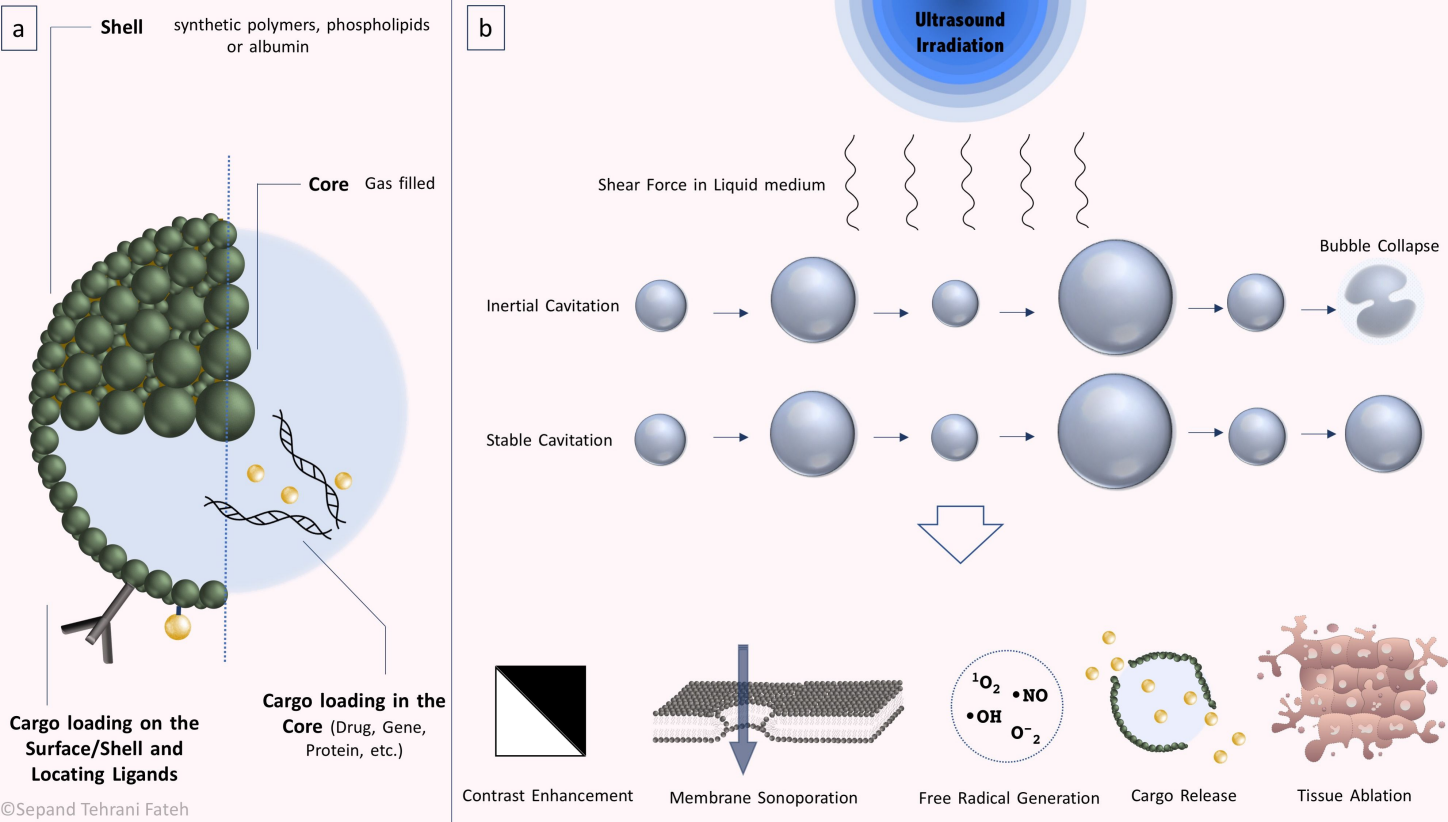
MB structure and mechanism of action. (a) MBs are gas-filled shell-coated particles that can be decorated with ligands or loaded with cargos. The cargo can also be loaded in the core of the structure. (b) MBs undergo inertial or stable cavitation under US irradiation, each of which can be used for specific applications. MB formation can enhance image contrast, facilitate cargo release, or cause tissue damage through different pathways.

Microbubbles have been widely employed as US-based medical imaging contrast agents [165–166]. They efficiently respond to US pressure waves and scatter the incident US energy due to their compressible gas-filled core. Therefore they can produce consecutive waves, amplify US signals, and eventually increase the image contrast [72,167]. In US-based drug and gene delivery systems, MBs have been used as carriers which can be loaded with a therapeutic agent and can be tracked or traced to the target site using low-intensity US imaging, and finally destroyed with a high-intensity burst of US. Thus, they can locally release the loaded drugs and enhance the penetration depth of the therapeutic agents into the targeted tissue via microstreaming and ARF [72,83,126,168]. Microbubbles can improve the efficacy of gene transfection, therapeutic agents, and anticancer drugs [139]. They can also be targeted to specific tissues through surface modification with different ligands [169–170]. Azmin et al. reviewed MB dynamics and the physical principles behind MBs, providing a theoretical basis for the development of MB-based theranostic systems [171]. Some articles have reviewed the application of MBs in theranostics [172–174]. The schematic illustration of the mechanism of action of MBs is presented in Figure 3.

Ultrasonication has been the major method used to actually produce MBs in the laboratory. The cavitation phenomena occurring as a result of US wave propagation shears the liquid medium causing MB formation. The manufacturing process can be carried out by two methods. Firstly, a batch sonication is performed in which the precursor material of the MB shell is sonicated in the presence of the inner gas to be encapsulated. Secondly, a continuous sonication is applied in which a continuous flow of both the inner gas and the shell precursor material are simultaneously sonicated in a uniform tank [156]. Microfluidic systems have recently been used as a method for MB production based on an interface between a liquid flow and a gas flow. T-junction and flow-focusing are the two major methods for microfluidic production of MBs. In the T-junction approach, the two flows (liquid and gas) are perpendicular to each other, whereas in the flow-focusing method, one flow is surrounded by the other flow when they emerge from a small orifice. Microfluidic systems are capable of producing multilayered MBs. Other advantages are the controllable size, adjustable shell and gas composition, uniformity of the bubbles, and their physical properties. On the other hand, the production rate of this system is not very high or efficient, which limits the translation from the lab to the clinic [175–176].

Microbubbles can be modified in order to improve their functionality, efficacy, and properties. Different parts of the MB structure can be utilized for cargo delivery. It is possible to create an oil layer inside the MB for cargo loading [177]. The surface of MBs can be loaded with cargo and also modified with specific ligands for targeting [178]. The use of MBs in combination with other types of NPs could also provide additional possibilities. These hybrids could enhance the accumulation, penetration, and uptake of nanomedicines [179–181]. These combinations have been used in theranostics for simultaneous imaging and drug delivery [182–184] or for multimodal imaging [185]. Microbubbles can enhance the endocytosis process by affecting cell signaling pathways [186]. One study showed that MBs, in addition to US irradiation, had a synergistic effect on triggering exocytosis leading to the release of extracellular vesicles [187].

Microbubbles were first developed as contrast agents and then were used in cargo delivery. Nowadays, they play a role as theranostic agents [178,188]. Many studies have shown that the concurrent use of MBs and US irradiation increases the efficacy of drug delivery or imaging contrast in cardiovascular diseases [189], cancer [190–193], infectious diseases [194–197], brain disorders [198–199], vaccines, and immunotherapy [200].

Escoffre et al. designed liposomal DOX-loaded MBs (DOX-liposomal-MBs) and assessed their effect on human glioblastoma (U-87 MG) cells under US exposure at a frequency of 1 MHz. In this study, thiol–maleimide was used to cross-link the DOX-containing liposomes onto the surface of the MBs. The in vitro results showed a four-fold enhancement in cell death using DOX-liposomal-MBs plus US in comparison with free DOX or DOX-liposomal-MBs without US. They concluded this was due to an increased intracellular uptake and release of DOX into cancer cells caused by cavitation events after US. They showed the uptake and accumulation of the drug in the cytoplasm and nuclei resulting from a disruption of the cell membrane, which was statistically significant when US was applied in combination with MBs. Although MB cavitation in an ultrasonic field has been extensively studied, the biophysical mechanisms leading to enhanced drug delivery are still a matter of debate [201].

Microbubble oscillations also result in a chemical effect on the cell membrane by inducing the formation of free radicals which can increase cell membrane permeability and the influx of Ca^2+^ ions [202]. Juffermans et al. showed that the catalase enzyme, a free radical scavenger, completely inhibited the Ca^2+^ influx at lower acoustic pressures (50 kPa). They then blocked the Ca^2+^-activated K^+^ channel (BKCa channels) using verapamil, a specific Ca^2+^ channel blocker, to investigate how a high acoustic pressure of 250 kPa increased cell permeability and Ca^2+^ influx. Interestingly, they observed that the blockage of the BKCa channels at 250 kPa led to a high depolarization of the cell membrane due to a large influx of Ca^2+^ ions [203]. They showed that the entrance of Ca^2+^ into the cytosol after employing the highest pressure (250 kPa) did not occur through BKCa channels. Unfortunately, they did not examine the effect of verapamil at the lowest US intensity of 50 kPa. However, they reported that the mechanical stress produced at the highest intensity led to the disruption and formation of specific pores in the cell membrane and extra Ca^2+^ entry which could not be inhibited by free radical scavengers or specific ion channel blockers. They concluded that the simultaneous entry of Ca^2+^ ions is an indication that the acoustic pressure leads to diffusion of Ca^2+^ ions through the cell membrane pores [204]. The fact that the intracellular Ca^2+^ ion concentration depends on the extracellular Ca^2+^ ion concentration supports this conclusion [202].

Dong and colleagues prepared plasma MBs by mixing plasma gas and surfactant using an emulsification process. These MBs released the loaded drug and also generated active free radicals (including nitric oxide and hydrogen peroxide) in response to US irradiation [205]. Microbubbles have also been employed as carriers of O_2_ into the tumor microenvironment. An oxygen-loaded lipid-coated preparation of MBs with mixed gas (O_2_/C_3_F_8_ 5:1 v/v) increases the PO_2_ of the tumor tissue almost six-fold compared with untreated tissue after exposure to US [206].

Aliabouzar et al. demonstrated enhanced proliferation and chondrogenic differentiation of human mesenchymal stem cells by applying lipid-coated MBs plus low-intensity pulsed US. After treatment, cell proliferation was increased by 40%, and the production of glycosaminoglycan and type II collagen was increased by 17% and 78%, respectively [207]. Liao et al. fabricated epidermal growth factor-coated lysozyme MBs responsive to US waves, which showed good antimicrobial activity, promoting neovascularization and significantly reducing the time needed for wound healing [197].

Several studies have been carried out to evaluate MBs plus focused US (FUS) for increasing blood–brain barrier permeability in order to enhance drug delivery to the brain. Different aspects of this method have been reviewed before [198–199]. Some studies have shown the application of MBs in sonothrombolysis. The presence of MBs enhances the efficacy of cavitation-induced US microstreaming in order to destroy blood clots in the brain [208–209].

Many publications have addressed the utilization of MBs in gene therapy or cell therapy [210–213]. Delivery of genes or immune-stimulatory materials to enhance cell-based immunotherapy via MBs could provide a promising approach to improve cancer immunotherapy [193]. Recently, Rinaldi et al. reported that sonoporation and MBs could enhance the transfection efficacy of the genes for TRAIL and p53 by 30–50% and activated the apoptosis pathway in liver cancer cells [213]. The positive charge of these MBs makes them potentially capable of interacting with negatively charged nucleic acids [211]. Researchers recently fabricated hybrid cationic MB–pDNA composed of DMAPAP. According to their report, the hybrid structure had similar acoustic activity to MBs and it was stable up to 30 min [210].

However, the application of MBs is challenging due to some inherent disadvantages. For example, the relatively large microsize (10 µm) of MBs restricts their efficient penetration into the solid tumor microenvironment, even with endothelial gaps ranging from 380 to 780 nm. Microbubbles have poor in vivo stability and a relatively short circulation half-life of 5–20 min due to inner rapid gas diffusion and the instability of traditional lipid shells [203]. In addition, MBs have a limited drug-loading capacity [214] and their surface is not easily modified with functional molecules to provide targeted drug delivery [83,215]. Upon injection, MBs will circulate for only a few minutes and then get stuck in the lungs [216]. Moreover, the MBs may cause irreversible damage to off-target normal tissues [171]. Therefore, MBs and US-triggered drug delivery may be restricted to tumor endothelial and cardiovascular targets. Fortunately, these limitations may be overcome by changes in the stiffness of the MB shell (fabricated from synthetic polymers, phospholipids, or albumin). Poly(vinyl alcohol) (PVA) has been used as a shell to improve physicochemical properties of MBs. PVA-based MBs show remarkable stability for several months. As a biocompatible component, PVA not only imposes no further toxicity to the biological system but also makes the conjugation of other components possible, enabling the delivery of hydrophobic drugs or DNA [217]. PVA also offers superior acoustic properties to the MBs. It has been shown that PVA-based MBs have better linear scattering performance than other polymeric-shelled MBs. The acoustic properties of these MBs, including backscattering, attenuation, and dispersion, also depend on the temperature [218]. Like many other NPs applied intravenously, PVA-based MBs are also prone to become uptaken by the reticuloendothelial system; however, they stay in the blood circulation long enough to act as an efficient contrast agent [219]. Some novel nanosystems that have been developed are composed of liquid PFCs, perfluorohexane (PFH), PFP, phosphatidylethanolamine, and halocarbons as probes for US molecular imaging applications and as carriers for drug/gene delivery [129,171,220].

#### Micelles

Micelles are one of the most useful types of nanocarriers for efficient drug delivery [221]. Micelles can be traced back to the discovery that certain surfactants formed particles with a size range of 10–200 nm when the concentration was increased [222]. Micelles are a colloidal dispersion consisting of amphiphilic molecules with hydrophilic tails oriented towards the surrounding water forming a shell and their hydrophobic heads (often composed of hydrocarbon chains) oriented towards the core of the structure [83]. The micelle core is formed based on van der Waals bonds [223]. Hydrophobic cargos can be located within the core of the micelles, while hydrophilic molecules can be attached to the surface of the micelles [221]. The hydrophilic part also plays an important role in structure stability and protects the micelles from external degradation or elimination [224–225]. Micelles can be fabricated from naturally occurring surfactants or from synthetic polymer components [221].

Micelles can also be used to carry small organic molecules, peptides, carbohydrates, monoclonal antibodies, and DNA or RNA aptamers [83]. The size of micelles is within a range of 5–100 nm depending on the type of the head group and the length of the alkyl chain [226]. Micelles can be assembled with different morphologies, such as spheres, rods, tubules, lamellae, vesicles, crewcut, star-like, flower-like, disk-shaped, toroidal and double-faced depending on the nature of the amphiphilic molecule, the solvent, and the temperature [224–226]. Non-spherical forms of micelles are not as stable as spherical shapes; however, a cross-linking in their structure may improve their stability and can potentially make stimulus-responsive micelles possible [225]. Some of the possible triggers for stimulus-responsive micelles include pH [227–228], temperature [229], enzymes [230], redox potential [231], light [232], US [233], electric fields [234], or magnetic fields [235]. Micelles can be fabricated combined with other NPs or biomolecules in order to have multiple features and functions including enhanced targeting, responsive particles, magnetic or fluorescent properties, all of which are important for theranostic applications [225].

When the concentration of the surfactant molecules or the polymer blocks is increased beyond a specific threshold, these components start to form the micelle structure. This is called the “critical micellar concentration” (CMC) [83]. Below the CMC value, the micelles will be remain dissolved in the medium [221]. Micelles that have a lower CMC are more thermodynamically stable and, in this sense, polymeric micelles tend to be more stable than surfactant micelles [221,224]. Using polymers with more hydrophobic blocks, a decrease in the hydrophilic block length and an increase in the hydrophobic chain length lead to overall increased micellar stability [225]. The CMC value for surfactant micelles is typically 10^−3^ to 10^−4^ M, while for polymeric micelles is 10^−6^ to 10^−7^ M [226]. Micelles also show kinetic stability in addition to thermodynamic stability and, therefore, allow sustained drug release [225]. The thermodynamic and kinetic stability of the polymeric micelles is important in order to preserve the integrity of the drug loading and prevent premature drug release prior to reaching the targeted tissue [222].

The naturally occurring surfactants used for micelles have included fatty acid alkyl esters of glycerol and phosphoglycerol esters. However, since the advent of polymeric micelles, most of the attention has been devoted to these new formulations [221]. The surfactants used in micelles can be divided into four categories: anionic (phosphates, carboxylates, sulfates), cationic (usually amine-containing surfactants), zwitterionic (phosphocholines and synthetic surfactants), and non-ionic (ethoxylate, glucosides) [221]. The use of polymeric micelles has led to their biological applications become more common due to their improved targeting ability, stability, long-term circulation, protein absorption, controlled and sustained drug release, higher molecular weight, slower dissociation rate, biodegradability, better penetration, higher drug loading capacity, and improved pharmacokinetic and pharmacodynamic profiles [224,236]. The lower CMC value of polymeric micelles compared to surfactant micelles is another reason that polymeric micelles are becoming more common in pharmaceutical formulations [237].

The components of polymeric micelles include diblock copolymers, triblock copolymers, and graft polymers [224]. Amphiphilic diblock or triblock copolymers are the most commonly used components [222]. The selection of polymers is based on biocompatibility, biodegradability, solubility, release rate, and hydrophobic core composition [225–226]. Polymeric micelles and their properties depend on the properties of the hydrophobic blocks and the solvent, the surface tension of the blocks in the solvent, interactions between block copolymers, temperature, and additives which are all discussed in the review [225]. The core of the polymeric micelles acts as a drug reservoir and the shell (corona) inhibits opsonization, aggregation, and slows down elimination within the body, in addition to providing better colloidal stability [238]. Pluronic copolymer micelles are one of the most frequently used types of micelles in many studies. Pluronic micelles are composed of triblock copolymers of hydrophobic poly(ethylene oxide) blocks and hydrophobic poly(propylene oxide) blocks [237]. Among the various types of Pluronic polymers, micelles constructed from Pluronic P105 have been used more often than other types as US-triggered drug delivery agents [237].

Many papers have described the release of different cargos from micelles after US exposure [239–241]. It has been suggested that the cargo release from micelles under ultrasonication is explained by cavitation processes. Cavitation and bubble formation and collapse cause a shear force on the micelles which leads to cargo release [242–243]. Moreover, ultrasonication is known to activate endocytosis and pinocytosis processes and cause perturbations in the cellular membrane, which together facilitate the uptake of the released cargos by the targeted cells [244]. The released cargos may be re-encapsulated after the end of US exposure [221]. In addition, hyperthermia caused by ultrasonication increases the overall destruction of the micelles and may also increase cargo release [245].

Wu et al. demonstrated the enhancement of chemotherapy through the utilization of both US alone and US-responsive micelles. Their results showed an increased accumulation of the intended drug and micelles within the sonicated cells and tissues. Moreover, they mentioned that the release of drugs from micelles was mostly dependent on the intensity of the US rather than on its duration [246]. Similarly, Rapoport et al. demonstrated that longer pulses with shorter inter-pulse intervals led to the occurrence of drug re-encapsulation, which could lessen the side effects of any excess drug that may be present in the environment [242].

Li et al. presented a novel preparation method of polymeric micelles, responsive to both HIFU and redox potential, as a potential nanocarrier system for the delivery of encapsulated pyrene as a model cargo [131]. This dual system contained a disulfide bond between biodegradable PEG and poly(lactic acid) (PLA) block copolymers as the central linkage, which could be cleaved in the presence of either HIFU irradiation or reducing agents, such as glutathione. The authors reported the collapse of cavitation MBs after applying HIFU irradiation in combination with glutathione treatment producing a solvodynamic shear force which led to site-specific scission of the disulfide bond, disruption of the micelles, and finally, irreversible controlled release of the encapsulated pyrene from the PLA and PEG block micelles [131].

Similarly, another group investigated the release behavior of poly(2-oxaline) micelles which differed in the composition of the copolymers loaded with dexamethasone as a cargo. They reported a 6–105% increase in the amount of drug release depending on the type of the copolymer, amount of the encapsulated drug, and duration of stimulation [247]. In another study, researchers fabricated amphiphilic hyaluronic acid micelles loaded with docetaxel. They reported that HIFU exposure expanded the diameter of the micelles, enhanced the release of encapsulated drugs through the disintegration of the micelles, and also increased the cellular uptake of the particles due to alterations in the permeability of cell membranes [237].

Micelles have also been used in sonodynamic therapy. Takemae et al. examined the synergistic effects of epirubicin-conjugated polymeric micellar NPs, which could be triggered by pulsed HIFU as a sonodynamic agent for cancer therapy. In this system, US irradiation caused ROS generation while the encapsulated drug could be protected from ROS due to the hydrophilic shell of the micelles, although it acted as a sonosensitizer itself. This method may affect cancerous cells in three different ways: mechanical and thermal effects of the US on the cells, the release of antitumor drugs, and ROS generation causing cell damage. They measured the concentration of hydroxyl radicals and superoxide anion after US irradiation. They stated that hydroxyl radicals could cause epirubicin degeneration. They reported a direct relation between hydroxyl radical generation and US irradiation intensity even at short durations. Moreover, although epirubicin was capable of generating superoxide anions under US irradiation, the micellar structure (NC-6300) could act as a sonosensitizer and produce even more superoxide anions. Hydroxyl radicals and superoxide anions caused apoptosis in cells at concentrations of 20 µM [248].

Similarly, Horise et al. demonstrated the use of micelles in sonodynamic therapy of various canine cancers, including chondrosarcoma, osteosarcoma, hepatocellular cancer, and prostate cancer. These researchers used NC-6300 micelles as antitumor sonosensitizers, which were filled with epirubicin and showed a synergistic effect with HIFU irradiation. The NC-6300 micelles accumulated in the tumor due to the enhanced permeability and retention (EPR) effect, where it efficiently generated ROS upon US stimulation. The dog with chondrosarcoma showed an 85% shrinkage in tumor size two weeks after sonodynamic therapy and was able to walk and run after the procedure, which was not possible before the procedure. The dog with osteosarcoma showed a modest reduction in the size of the tumor; however, the severity of the pain was lower. In hepatocellular cancer, the tumor continued to grow but on a slower rate compared with the growth rate before sonodynamic therapy. In the dog with prostate cancer with a calcified mass and lung metastasis, the mass and the metastasis disappeared after the procedure. The disappearance of the calcified mass might be due to HIFU irradiation, even though the power was lower than that of conventional HIFU therapy. They also hypothesized that although the disappearance of the lung metastasis was unanticipated, it may be due to an immune response subsequent to sonodynamic therapy. Their results supported the potential of using micelle-based sonodynamic therapy in cancer treatment. Moreover, their system was capable of enhancing US imaging contrast via MB formation during US irradiation [249].

Kang et al. prepared NO-donor-loaded micelles, which could be triggered by HIFU irradiation. The NO-donor was 1,3-bis-(2,4,6-trimethylphenyl)imidazolylidene nitric oxide (IMesNO) that released NO gas via thermolysis. These IMesNO-loaded micelles enhanced the accumulation of the drug in the tumor site through the EPR effect. Ultrasound irradiation first caused NO generation, which led to vasodilation and a subsequent further increase in the drug-loaded micelles accumulated in the tumor vessels [250].

#### Liposomes

Liposomes are vesicle homologues of cellular membranes composed of two enclosed layers of phospholipids. Phospholipids are amphiphilic molecules with a hydrophobic long hydrocarbon chain(s) and a hydrophilic head. Phospholipids are the main component of liposomes; however, cholesterol and other polymeric blocks can be present [251–252]. Due to the nature of phospholipids, they self-assemble in the presence of water and form various structures. Vesicles are the most stable structures as the bending of the lipid bilayer and vesicle formation reduce edge interaction energy which arises from the partial exposure of nonpolar hydrocarbon chains to the aqueous phase. The preparation methods, lipid type and charge, lipid composition, surfactant, organic solvent, and ionic strength of the suspension medium determine the physical properties of the liposomes. Liposomes can be prepared in a multilayer formation, which is dependent on the preparation method. Reverse phase evaporation, detergent depletion, lipid hydration, freeze-thawing, and alcohol injection methods are among the most conventional methods for preparing vesicles [251,253]. Moreover, small and uniform liposomes are desirable and microfluidic-based preparation methods allow for a scalable production of such liposomes [254]. Liposomes can entrap both lipophilic and hydrophilic compounds due to their amphiphilic nature. Hydrophilic compounds can be localized in the inner space of liposomes, whereas the lipid bilayer can enclose lipophilic compounds [255]. Liposomes can be engineered to become stimuli-responsive. This feature would add a desirable control over cargo release in intended sites. Ultrasound [256], light [257], heat [258], and pH [258] can trigger the release of cargo from liposomes. Liposomes can also be combined with other materials to become responsive to magnetic and electrical fields [258]. These nanoparticles are typically considered biocompatible and pharmacologically inactive with minimal toxicity; however, their toxicity is related to the exposure time, dose, surface properties, cholesterol content, charge, and degree of saturation and length of fatty acids [252]. Liposomes can enhance drug delivery efficacy. The therapeutic index is generally increased by the encapsulation of drugs into liposomes with a longer blood circulation half-life, leading to passive targeting through the EPR effect of solid tumors with leaky vasculature [259]. However, their phagocytosis in the blood circulation is still challenging [252]. Liposomes can also be targeted via surface ligands in order to maximize the efficacy of cargo delivery and minimize systemic side effects [260–262].

**Figure 4:**
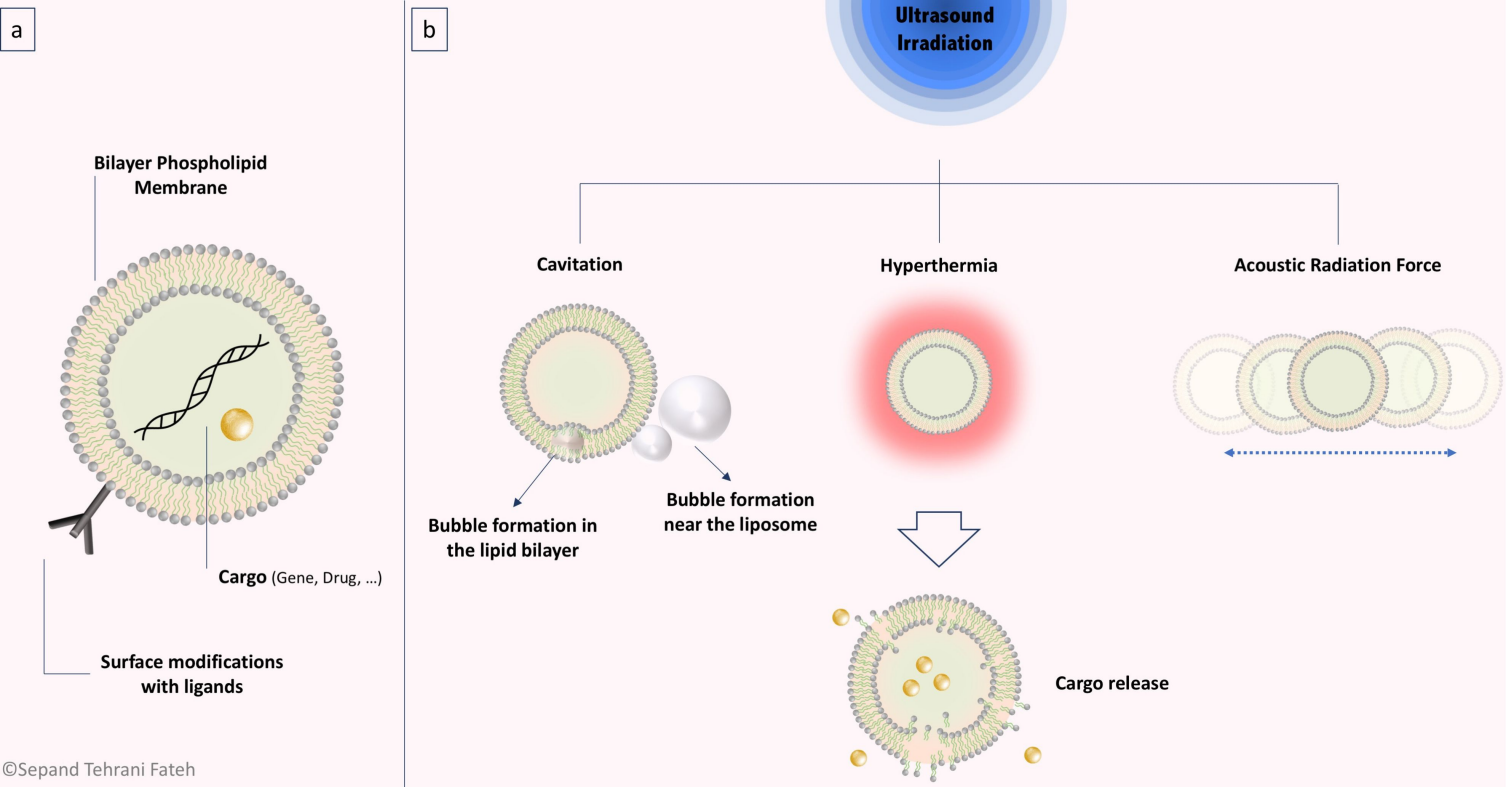
US-triggered liposomes. (a) Liposomal structure with phospholipid bilayer membrane and an aqueous core. Liposome surface can be modified with different ligands for more biological functions and the desired cargo can be loaded into the core of the structure. (b) Mechanism of action of cargo release from liposomes under US irradiation. Cavitation in the lipid bilayer or near the liposome, hyperthermia, and ARF are possible mechanisms that could cause a decrease in membrane integrity and lead to cargo release.

**Conventional liposomes.** Based on several studies, US is able to initiate drug release from liposomes [263], even if the main primary mechanism of drug release is not completely understood. It has been assumed that different mechanisms may take part in the release and may be influenced by the specific US parameters and chemical structure of the liposomes. The probable mechanisms for drug release from these structures involve cavitation, thermal effects, and acoustic streaming and these mechanisms may be somewhat overlapping (Figure 4).

As mentioned above, cavitation describes the creation and abrupt collapse of a vapor bubble near or at the lipid bilayer membrane of the liposome. The cavitation process in DDS must be sufficiently intense in order to disturb the membrane and release the liposomal contents. Recent studies have proposed that cavitation triggered by low-frequency US is able to disrupt liposomes and stimulate the release of encapsulated cargo [264]. Schroeder et al. [265] showed that low-frequency (20 kHz) US was used to trigger the release of three different encapsulated drugs including methylprednisolone hemisuccinate, doxorubicin, and cisplatin. An efficient release (≈80%) of drugs from liposomes was achieved by means of US exposure in periods of up to 180 s, and this did not depend on the type of drug or on the method of drug loading. This result was ascribed to the long-lasting deterioration of ≈20% of the liposomes and creation of transient pores in the remainder. In another study, Somaglino et al. [266] reported the release of DOX from liposomes in the presence of high-frequency (1 MHz) pulsed US to avoid substantial temperature changes from US exposure and take advantage of the cavitation effects. Increased temperature is another possible mechanism to explain cargo release from liposomes after US exposure. If the thermal effects of US were responsible, the rate of drug release would be less abrupt and the permeability of the liposomes could be increased if the local temperature rose above the lipid phase transition temperature (Tm) [267–268]. Needham et al. [269] tested a temperature-sensitive liposome (TSL) formulation in which the Tm was just above physiological temperature (37 °C), producing drug release with trivial hyperthermia. The DOX release was more efficient from the gel-like condensed liposome state (ordered packing) compared to the more fluidic state (disordered packing). The mechanism was attributed to the increased permeability of the boundary defects during phase transition. Similarly, Dromi et al. [270] used pulsed HIFU to trigger TSLs in order to produce rapid drug release and local DOX delivery to tumors. A more recent study showed that acoustic streaming could also play a role in efficient drug release from liposomes. Oerlemans et al. [271] demonstrated that the nature of the encapsulated cargo, either hydrophilic or hydrophobic, is a determining factor in their release in response to a certain mechanism of action of ultrasonication. It has been observed that the release of hydrophobic cargo was attributed to non-cavitational and non-thermal effects of US. It has been hypothesized that the radiation force-induced collisions of particles during US exposure could cause the release of hydrophobic cargo from the lipid bilayer [41].

Based on the studies of Garcia-Sabaté, solid NPs trapped in liposomes when exposed to US waves could cause movement and break down the lipid membrane. This response to US was due to US-induced mechanical effects rather than US cavitation, and these novel liposomes were more stable than those encapsulating a gas core [272]. Xiaoping Zhan and coworkers [273] have utilized poly(lactic-*co*-glycolic acid) (PLGA) liposomes plus high-frequency US (>1 MHz) to trigger the release of mitoxantrone on demand. In their study, when the liposomes were stimulated by US, the lipid membranes were ruptured and the mitoxantrone molecules were released.

Noninvasive image-guided drug delivery has recently been reported using TSLs plus HIFU for the delivery of anticancer therapeutics. Ultrasound-induced hyperthermia could significantly modify the permeability of the tumor vasculature and boost nanoparticle uptake. In 2018, researchers described TSLs that encapsulated topotecan (Hycamtin^®^), a chemotherapeutic agent which could be monitored by an increase in its intrinsic fluorescence when it was released in the tumor [144]. In this work, a new DDS based on TSL labeled with both MRI and a near-infrared fluorescence (NIRF) imaging agent was designed, which permitted liposome tracking using both modalities. The localized mild hyperthermia (<43 °C) was used to increase the nanoparticle concentration within the tumor by improving local blood flow and reducing interstitial tumor pressure. This approach increased the uptake of NPs up to ≈400 nm in diameter. Focused ultrasound (FUS) caused deep and localized hyperthermia in a controlled manner as measured by MR thermometry. This could improve the accumulation of NPs in the tumor and activate drug release from the carriers. Near-infrared (NIR) imaging showed that the selective accumulation of TSL within the tumors was achieved. Mild FUS-induced hyperthermia (3 min at 42 °C, 30 min post i.v*.* injection) greatly enhanced the uptake of the TSLs. The colocalization of topotecan fluorescence emission was also observed immediately after application of FUS, indicating rapid US-triggered drug release. It was proposed that the TSL accumulation and parallel topotecan release was increased by a second mild hyperthermia treatment applied 1 h after the first. Moreover, MRI was carried out in vivo to verify the enhanced TSLs uptake due to the FUS treatment.

Liposomes can be functionalized with antibodies and ligands for targeting purposes. The strategy enhances the therapeutic or diagnostic efficacy and reduces the possible systemic side effects. In a study, calcein and doxorubicin‐loaded liposomes were functionalized with the monoclonal antibody trastuzumab for targeting human epidermal growth factor receptor 2 (HER2) positive breast cancer cells. These cancer cells overexpress the HER2 receptor on their surface and the probability of an interaction between trastuzumab-functionalized liposome and these cells would be higher than an interaction with other healthy cells. Low-intensity focused US (LIFU) was used to trigger the release of drugs from liposomes. Trastuzumab-functionalized liposomes showed higher cellular toxicity and higher drug uptake by the HER2-positive cell line and the addition of LIFU further improved the therapeutic outcomes [260]. In a similar study, calcein‐loaded liposomes functionalized with transferrin were used for the targeting of HeLa cells. Similarly, a synergistic effect between administration of LIFU and the targeting properties increased the therapeutic efficacy of the treatment [261]. A study on the concurrent use of LIFU and calcein‐loaded liposomes functionalized with human serum albumin for site-specific breast cancer therapy demonstrated similar results [262]. These studies highlight the synergistic effect between administration of LIFU and targeting properties for better therapeutic outcome.

Recently, researchers [274] proposed a novel therapeutic strategy consisting of the magnetic accumulation of ultra-magnetic liposomes (UML) followed by HIFU to trigger the release of an antivascular agent which could be monitored by MRI. They encapsulated combretastatin A4 phosphate (CA4P), a vascular disrupting agent, in the core of an UML in order to prepare CA4P-loaded thermosensitive CA4P-UML. CT26 murine colon tumors were studied as a model to investigate the effects of this system. It has been revealed that the combined treatment had additional benefits after 24 h of treatment and a 150-fold enhancement in the antitumor response compared with chemotherapy was observed.

**Echogenic liposomes.** Large echogenic liposomes have been used as targetable contrast agents for US imaging. They are liposomal particles with submicron diameters that include a gas or a gas-generating precursor molecule in their central core [275–280]. Due to thermodynamic reasons, a gas incorporated within a liposome can be assumed to behave like a hydrophobic drug which is located between the two monolayers of the liposomal bilayer, or else as a monolayer-covered gas bubble within the aqueous interior compartment of the liposomes [281]. The gases used are typically air or nitrogen, or else bio-inert heavy gases, such as PFCs or sulfur hexafluoride. The composition of the inner core gas and the shell material predominately affects the physicochemical properties of the echogenic particles, such as physical stability, biological half-life, and echogenicity within the human body. Heavy gases decrease the gas diffusion rate and slow down the leakage of gas from the inner core of the liposomes into the surrounding fluid; thus, the lifetime of the MBs in the bloodstream can be prolonged [282]. The efficiency of gas encapsulation relies on the properties of the compressed gas and the lipid shell [283], for instance: gas diffusion across the lipid shell [284–285], the thickness of the lipid shell, size of the MBs [286–287], and the presence of human serum or albumin in the medium [288].

Three basic echogenic liposomal structures have been prepared using different methods. The first structure consists of two compartments in which the smaller section contains the gas and is surrounded by a monolayer, while the larger compartment holds the aqueous phase. These echogenic liposomes can be produced via the freeze-lyophilization process [275,289] or the pressure-freezing method. The second configuration is composed of a monolayer-covered gas bubble within the aqueous core of the liposome [290]. These bubble liposomes are modified with PEG-liposomes and can be prepared via the reverse phase evaporation procedure. The liposomal components are introduced into containers supercharged with perfluoropropane gas and then sonicated in a bath sonicator [291]. The third and last structure is a hybrid complex in which conventional liposomes are conjugated to stabilized gas bubbles through a biotin–avidin linkage [292].

Depending on the physicochemical properties of the echogenic particles, four distinct processes could occur after US irradiation: (1) the gas diffuses out from the echogenic liposomes as they steadily reduce in size, (2) the US irradiation can cause shell defects permitting the gas to be released, (3) the echogenic particles break down into smaller particles, or (4) the echogenic particles are rapidly eroded, so the shell material breaks away from the gaseous inner core [280]. The response of echogenic liposomes to ultrasonication is influenced by the liposomal composition, the encapsulated gas, and the US parameters [281]. The flexibility of liposomes plays an important role in their fate after ultrasonication and it affects the liposome response to US. Particles with hard or rigid shells would fracture after exposure to sufficiently intense US, while lipid-encapsulated MBs can oscillate and, even during the US pulse, could promptly re-assemble [293–294]. Therefore, upon application of US, liposomes with a gaseous core may expand more than 10-fold beyond their initial surface area before destabilizing and coalescing [293].

Echogenic liposomes are able to capsulate hydrophobic drugs; however, since these drugs have higher solubility in the lipid bilayer than in water, they should be relatively resistant to be released from liposomes by US. Moreover, the drug may remain in the lipid fragments of the disrupted liposome. To overcome this issue, hydrophobic drugs could be changed to become more hydrophilic by combining them with cyclodextrins, which have a hydrophilic external surface and make a complex with hydrophobic drugs through their hydrophobic binding pocket [295].

The conjugation of liposomes with MB is a possible way to prepare US-responsive liposomes. Liposome–MB conjugates have recently been introduced as US-responsive platforms for cancer therapy. Nonetheless, they are limited by their size for good tumor penetration and have been investigated only as passive carriers. In 2018, submicron-sized (756 ± 180.0 nm), phosphatidylserine-based paclitaxel–liposome–nanobubble conjugates (PSPLBC) were reported by Banerjee et al. [296] to exert a pro-apoptotic anticancer effect and also to allow for image guidance. The drug release from the PSPLBC was activated through US-mediated cavitation. In vitro experiments showed a 10-fold increase in cellular internalization compared to a control sample. Moreover, the strong synergism between polystyrene (PS) and paclitaxel (combination index, CI < 0.1) explained the high antitumor efficacy both in vitro and in vivo (98.3 ± 0.8% tumor growth inhibition). In another study [297] from Banerjee and his team, they reported the preparation of submicron-sized (528.7 ± 31.7 nm) nanobubble–paclitaxel liposome complexes for US imaging and US-responsive drug delivery in cancer cells. The paclitaxel entrapment efficiency was 85.4 ± 4.39%, and the 200 nm-sized liposomes efficiently bound (conjugation efficiency ≈98.7 ± 0.14%) to the nanobubbles to form these complexes. The cellular uptake was increased by 2.5-fold compared to the liposomes alone after US irradiation. This has increased the therapeutic activity of the drug by more than 300-fold. Moreover, nanobubbles were shown to possess better echogenic stability compared to the commercial US contrast agent called SonoVue.

Yang et al. [298], described a 2,2’-azobis[2-(2-imidazolin-2-yl)propane] dihydrochloride (AIPH)-loaded liposome (Lip-AIPH), which could instantaneously produce gas bubbles and also yielded a high concentration of ROS under US irradiation. In vivo experiments showed that the production of gas and free radicals did not depend on the oxygen concentration. Moreover, this system could be used for enhanced sonodynamic therapy in a hypoxic tumor microenvironment. In order to study the effect of the gas MBs, MCF-7 cells were sequentially treated with Lip-AIPH and US irradiation and followed in real time by confocal microscopy. The number of gas bubbles surrounding the cells was increased as the US irradiation was prolonged. Finally, the cells lost their regular morphology and progressively died after US treatment due to the collapse of the gas bubbles through cavitation generated by the US shock.

Liposomes can also be filled with gas to become US-responsive. Ezekiel et al. fabricated echogenic 5-fluorouracil-encapsulated crude soy liposomes which were filled with argon. Administration of LIFU could release approximately 65% of 5-fluorouracil. The inner core gas is responsible for the US-responsive behavior as it expands subsequently to US exposure and liposome burst. This system can reduce the systemic toxicity (especially against bone marrow) and improve the therapeutic index [256].

In a novel study, researchers extracted exosomes from bovine milk and used them as echogenic exosomes for ultrasonography with enhanced contrast. Exosomes are naturally secreted extracellular bilayer vesicles from cells for communication and biomolecule delivery purposes. Their lack of toxicity and minimal immunogenic response give them an advantage over conventional liposomes. These echogenic exosomes showed significant linear and nonlinear scattered responses and can be used as effective ultrasound-responsive drug delivery systems [299].

#### Niosomes

Niosomes are bilayered non-ionic surfactant vesicles analogous to liposomes [300]. These bilayered vesicles can be either uni-layered or multilayered. Multilayered vesicles take the form of concentric vesicles located within each other [301]. The non-ionic surfactants used in niosomes are amphiphilic structures, such as alkyl ethers, alkyl glyceryl ethers, terpenoids, polysorbates, and polyoxyethylene ethers. These prevent vesicle aggregation and a transition from the gel state to the liquid phase which makes the niosomes less leaky [302].

The fundamental mechanism of niosome formation is similar to that of liposomes. The self-assembly of amphiphilic compounds leads to the formation of the vesicle structure. However, administration of external energy would facilitate this process [303]. Appropriate mixtures of surfactants and charge-inducing agents are required for thermodynamically stable niosomes. Monomer concentration, hydration temperature, time of hydration, pH of the hydration medium, cosurfactant, cholesterol, aqueous interlayer, lipid chain length, chain-packing, membrane asymmetry, and the nature of the drug are the most important factors that must be taken into consideration for the preparation of niosomes with desirable properties [303–304]. Physicochemical properties and the pharmacokinetics of the vesicles depend on the preparation method and this requires attention. Some of the synthesis methods of niosomes include thin-film hydration, ether injection, sonication, reverse phase evaporation, freeze and thaw, heating, and dehydration/rehydration [304]. In contrast to the aforementioned bulk methods, the preparation of niosomes with microfluidics would lead to more uniform niosomes with specific sizes [305]. The decision on the type of preparation method can be made based on the desired entrapment efficacy, size, preferred materials, drug-loading strategies, uniformity, and number of layers [304].

Niosomes are classified based on three factors, which are their intended function, method of preparation, and vesicle size. The main types of niosomes are based on the number of layers and size (i.e., multilamellar vesicles (MLV), large unilamellar vesicles (LUV), and small unilamellar vesicles (SUV)) [306]. Most niosomes are in the submicron size range. The particle size of SUVs is approximately 10–100 nm, LUVs are approximately 100–3000 nm, while MLVs are larger, approximately 5 micrometers. Some giant vesicles have also been reported [301].

Niosomes attracted attention due to the disadvantages of liposomes. More chemical stability, osmotic activity, longer shelf life, simple surface modification, less toxicity and more compatibility, biodegradability, and less immunogenicity are among the advantages of niosomes over conventional liposomes [304]. Osmotic activity, long storage period, controllable characteristics, and a relatively simple production process are other advantages of niosomes [306]. However, stability and cargo leakage also occur in niosomes similarly to liposomes [304,307]. Niosomes can be loaded with hydrophilic or lipophilic drugs or both kinds of drugs at the same time [302,306]. The morphology, vesicle size, vesicle charge, encapsulation efficacy, stability, permeability, and release profile are some parameters to be considered in drug delivery applications [308]. Niosomes have been used in cancer chemotherapy [309], HIV/AIDS treatment [310], vaccine and antigen delivery [311], pulmonary delivery [312], transdermal delivery [313], and in the delivery of proteins and peptides [314]. Niosomes can be administered through different routes, including parenteral, transdermal, oral, ocular, and pulmonary [303].

Hood et al. investigated the use of US to trigger drug release from niosomes. They encapsulated carboxyfluorescein as a model drug and measured the concentration of carboxyfluorescein in both the surrounding medium and niosomes after sonication. According to their results, the encapsulated carboxyfluoresceine decreased by two-fold while it increased by 10% in the solution, leading to the conclusion that the drug could cross the membrane without significant destruction of the niosomes or altering their size distribution [315]. In another study, researchers encapsulated Plai oil (a natural essential oil) inside niosomes and demonstrated synergistic effects of niosomes plus US irradiation for anti-inflammatory activity in comparison with a control group. They proposed that the drug release was caused by cavitation [316]. Hyperthermia could also be a possible mechanism of drug release from niosomes after exposure to US. Tavano et al. prepared thermosensitive niosomes and investigated the drug release behavior at temperatures of 25, 37, and 42 °C. They showed that the release was better at 42 °C and suggested that US-induced hyperthermia could be important [317].

#### Nanoemulsions (nanodroplets)

Nanoemulsions or nanodroplets are kinetically stable but thermodynamically unstable dispersions composed of two immiscible liquids, in which one liquid forms suspended spherical droplets within the other liquid [318]. Nanoemulsions have a core of nonpolar material, with a size <500 nm, suspended in a polar environment and is stabilized due to constant Brownian motion [318–320]. Nanoemulsions are usually oil in water (o/w) or water in oil (w/o), but can also be water in oil in water (w/o/w). The dispersed droplets of water and immiscible liquids need to be stabilized using emulsifying agents [319–320]. Emulsifying agents are amphiphilic surface-active molecules or surfactants which can reduce the interfacial tension between two immiscible liquid phases of oil and water by preferential adsorption at the interface [320]. Emulsifiers act by stabilizing the oil/water interface. Surfactants, phospholipids, amphiphilic proteins, polysaccharides, and other synthetic or natural polymers can all act as emulsifiers [320]. The components of NEs are oil or lipid, surfactant or co-surfactant, preservatives, antioxidants, or chemoprotectants [319]. Surfactants, albumin, polymers, and lipids can all be used as a component of the NE shell [321].

Nanoemulsions cannot be spontaneously formed without the introduction of some source of energy [322]. The production methods of NEs can be divided into high-energy and low-energy methods. In high-energy methods, a coarser emulsion known as the premix is treated with an external energy in order to reduce the size of the droplets through the action of a shear force [318]. The amount of shear force directly applied influences the droplet size and the presence of the surfactant decreases the required shear force [319]. As the droplets break down into smaller particles, the surfactants cover the newly produced surfaces and stabilize the newly formed droplets [318]. High-speed homogenization, high-pressure homogenization, ultrasonication, and microfluidics are some of the high-energy production methods for NEs [318]. High-energy emulsification uses energy to rupture the droplets in the presence of a surfactant which reduces the interfacial tension [323]. On the other hand, low-energy methods are based on the phase transition of emulsion systems due to changes in the temperature or composition of the system [318]. Low-energy methods involve a very low interfacial tension [323]. Spontaneous emulsification and phase inversion methods are two other kinds of low-energy methods for NEs production [319].

Nanoemulsions are prepared with a low surfactant concentration, which makes them prone to thermodynamic instability. Considerable external energy is required to reduce the size of the droplets down to the nanoscale, which makes them more kinetically stable. A low concentration of surfactant produces NE with less toxicity [318]. The relative viscosity parameters of the two phases, the type of oil, and the volume fraction are the parameters that influence the size of the droplets [319]. The particle size has an effect on their appearance, stability, optical properties, rheology, release profile, and bioavailability [324]. The forces between the droplets decrease as the size of the droplets become smaller [324]. Smaller NEs have more stability against gravitational separation, flocculation, and creaming due to the increased Brownian motion compared to larger NEs [320].

Emulsions generally degrade via coalescence, which results from collisions between the droplets and by Ostwald ripening [323]. It has been shown that Ostwald ripening is the major mechanism of destabilization in the case of NEs with a high surfactant concentration. However, when the droplets are not fully coated with surfactants, coalescence becomes more dominant. Hence, the likelihood of these mechanisms is directly related to the method of preparation [323,325]. Particle charge is also relevant for the stability and functional activity of NEs. The electrostatic charge is due to the absorption of mineral ions, ionized emulsifiers, or charged biopolymers around the surface of the NEs [324]. The properties of NEs have been reviewed in [326–327].

If the liquid lipids and surfactants are correctly selected, the NEs have the ability to stabilize large amounts of hydrophobic drugs with a high drug loading in the oil core of the nanosystem acting as the drug reservoir [320,322,328]. Conventional cargo release from NEs is explained by the Fick’s first law and may be prevented or controlled via grafting other polymeric components onto the surface of the NEs. Surfactants can also modulate the kinetics of drug release [319]. Both the physical parameters and the chemical composition of the NEs influence the properties, including cell uptake, drug release kinetics, clearance, and toxicity [320]. Nanoemulsions can undergo direct paracellular or transcellular transport leading to increased drug bioavailability [319,329]. NEs have been used in drug delivery applications with different administration routes, including intranasal, oral, ophthalmic, transdermal, topical, or parenteral [330]. Some NE-based drugs have reached the pharmaceutical market and are commercially available, while others are still at the preclinical or clinical trial stages of drug development and are on a waiting list for approval [320,331].

Many studies have reported the synergistic effects of US irradiation and NE-based drug delivery or enhancement of imaging contrast [332]. These NEs can be modified or hybridized with different components to improve the properties and provide multifunctionality [130,333–337].

An external stimulus such as FUS can trigger the liquid-to-gas transition in the NEs. This point of transition is known as the vaporization threshold. Above this limit, bubbles will be formed from the droplets in response to US irradiation. Shpak et al. explain this as a superharmonic focusing of acoustic energy, which causes a spot of negative pressure that spreads throughout the whole liquid volume [338]. In other words, the droplet-to-bubble transition of NEs under US exposure increases the interior volume of the vesicle, which then leads to vesicle rupture and drug release [339]. Nanodroplets have been proposed as an alternative to gas-filed MBs and might be superior in US therapy compared to gas-filled MBs. Microbubbles can be formed in the desired location via US application which produces negative pressure by the vaporization of droplets, known as ADV [115]. Moyer et al. demonstrated that the pressure required is related to the size of the droplets, type of PFC, and the pressure and temperature of the medium. The survival of the particle in the gas or liquid state is also related to these parameters. They further stated that NDs could also improve thermal delivery at the acoustic focus while avoiding overheating outside the focus, which is actually the opposite of how MBs function [115]. The generated MBs can also enhance the contrast of US imaging due to an increased backscattered signal [340].

**Figure 5:**
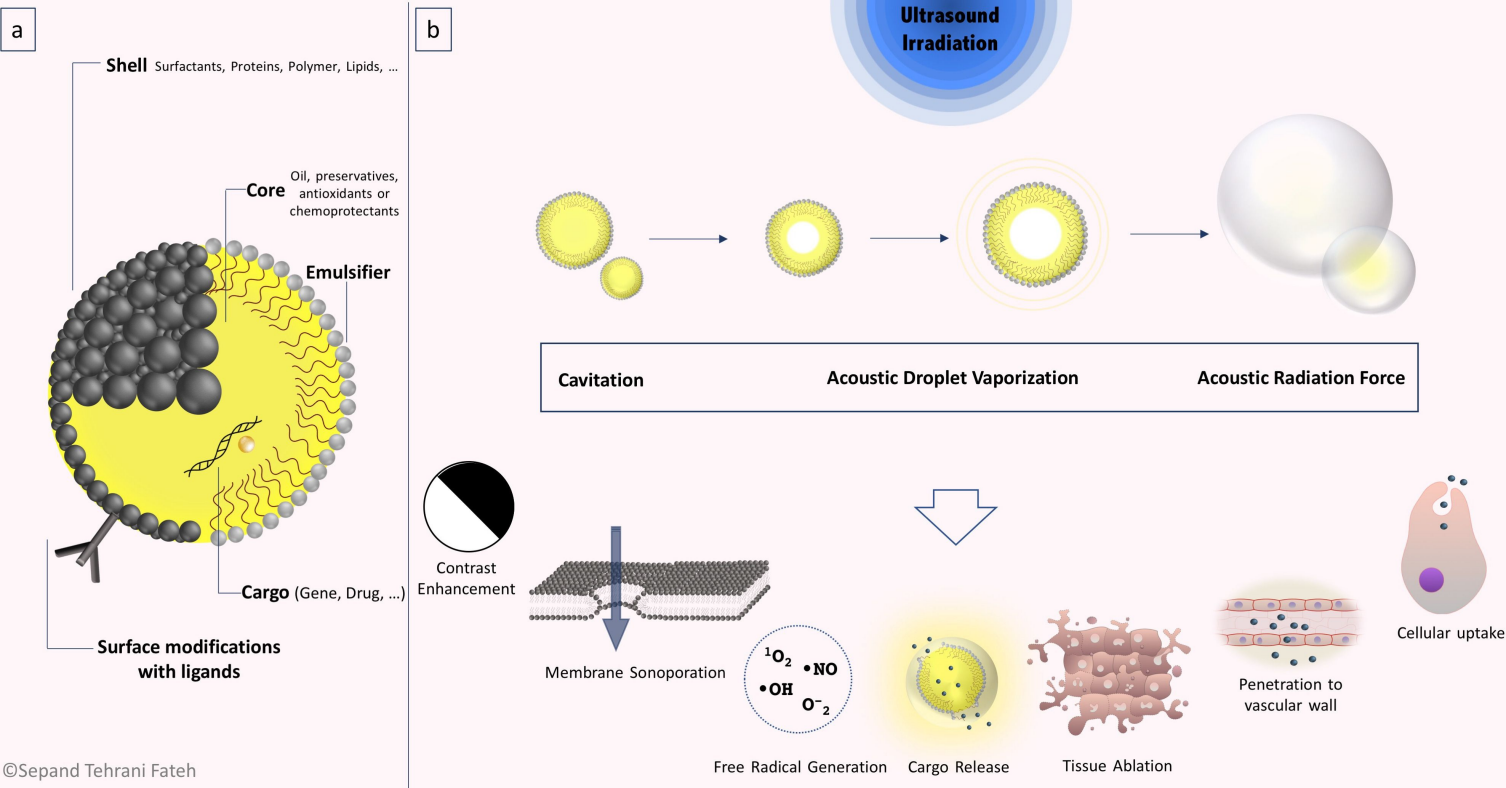
Structure and mechanism of action of NEs. (a) Nanoemulsions are composed of a core of hydrophobic liquid, stabilized via an emulsifier in an aqueous medium, which can be coated with a shell decorated with ligands. (b) Nanoemulsions are related to MBs and could be considered as a precursor of MBs. Under US irradiation, NEs can transform into bubbles. This phenomenon is explained by cavitation and ADV. The acoustic radiation force explains some of the biological effects of these particles.

Many papers have been published by Rapoport and colleagues in the field of US-triggered NEs. Some of these papers provided fundamental knowledge about NEs and US-triggered drug delivery or contrast enhancement [133,135,341–342]. Rapoport et al. developed a theranostic system based on NEs which converted into MBs at physiological temperatures. They noted that different sizes of MBs each had a specific role in the process of drug delivery and diagnosis. Nanobubbles and initial micron-sized bubbles are useful for drug delivery and US-mediated enhancement of cell uptake, while larger MBs provide strong contrast in ultrasonography [341]. In another study, the same researchers showed the synergistic use of drug-loaded NEs and US irradiation. It is worth noting that the NEs without any drug loading combined with US had no therapeutic effects [133]. They also synthesized perfluoro-15-crown-5-ether coated NDs as a drug carrier and contrast agent. According to their results, these particles were more stable, had a longer residence in the circulation, and passively accumulated in the tumor site compared with uncoated NEs. Acoustic droplet vaporization and cavitation may be the possible mechanisms for drug release and contrast enhancement [135]. The threshold for vaporization decreased with increasing US frequency and sonication time. As the size of the droplets decreased, the vaporization threshold increased. In smaller NEs, the Laplace pressure may increase the boiling point. This effect is caused by the surface tension at the interface between the droplet and liquid. Studies have shown that the ADV threshold is lower than the inertial cavitation, suggesting that the droplet-to-bubble transition occurs prior to inertial cavitation [343]. Similarly, Zhong et al. stated that the frequency-dependent drug release suggested an uncaging mechanism and not a significant thermal mechanism. Moreover, no evidence of cavitation was observed [344]. In contrast, Gao et al. suggested that the uptake of particles by cells may be attributed to the cavitation process [335]. Liu et al. stated that there might be some cell damage due to this cavitation process after LIFU irradiation [345]. Crake et al. used combined passive acoustic mapping and magnetic resonance thermometry for monitoring US-mediated cavitation-enhanced tumor ablation via NEs. This study also provided further evidence for the ability of phase-shifting NEs to promote cavitation-enhanced lesion formation [346]. The phase change threshold is dependent on US frequency, pulse duration, and droplet temperature [130]. Xu et al. suggested that by using dual-frequency FUS instead of single-frequency sonication, the vaporization of the droplets and the inertial cavitation could be controlled [347]. It has been demonstrated that low-boiling-point phase-changing NDs can act as US contrast agents by sonoporation without any significant adverse cellular effects. Moreover, by modifying the pulse length, these effects could be precisely controlled. However, further investigations are needed before in vivo use is justified [348]. Nanoemulsions may be used as inertial cavitation nuclei for the improvement of sonoporation efficiency. The details of the relevant parameters are reviewed in [349]. Sheeran et al. showed that it was possible to produce phase-shift droplets directly by condensation of commercially available MBs with a decafluorobutane core [350]. Different aspects of NEs are shown in Figure 5.

Low-frequency ultrasonication leads to a more effective bubble formation. In order to obtain smaller particles, higher energy and more intense US waves are required. In the case of bubbles filled with PFH, a higher frequency of US was needed compared with PFP, which were 476 and 20 kHz, respectively [351]. Studies have shown that the intracellular delivery and aggregation of NDs reduced the required US pressure for vaporization in order to induce cytotoxicity [352].

Cao and colleagues have investigated the drug release behavior of NEs combined with LIFU. They categorized the steps that are involved in the drug release profile. A sharply increasing curve of drug concentration at the start of the irradiation is caused by LIFU, and intra-tumoral accumulation and tissue distribution of the drug are enhanced with a second LIFU exposure [353]. Zhong et al. fabricated polymeric PFP NEs as drug carriers. They reported that the use of longer hydrophobic blocks of emulsifying polymers enhanced drug loading capacity. Moreover, they showed the versatility of this system which could load drugs with a wide range of properties. The drug release efficacy, clearance kinetics, and biodistribution were relatively independent of the properties of each individual drug, whereas the drug loading and drug uncaging were strongly affected by the drug hydrophobicity [344]. Baghbani et al. used various polysaccharide-coated NEs as US-triggered drug carriers and contrast agents. These particles showed a longer circulation half-life, better biodistribution, and no evidence of hemolysis [354–356]. Nanoemulsions can be coated with a silica shell which provides more stability, gives a stronger echo signal and contrast enhancement, and enables higher drug entrapment efficacy and targeting ability after decorating the silica shells with specific ligands [357]. Polydopamine has also been used for stabilizing NEs and rendering them more biocompatible [358].

Ultrasound can be used to increase the penetration of NEs into the vascular wall. It has been shown that continuous US was more effective than pulsed US with the same energy. The radiation force may be the mechanism for the conversion of NEs into bubbles because no significant increase in temperature was observed, which disproved the involvement of thermal effects in this process [359]. Nanoemulsions have also been used for delivering drugs to the brain since the permeability of the blood–brain barrier can be increased by the vaporization process occurring after US exposure. Wu et al. compared octafluoropropane (OFP) and decafluorobutane (DFB) for this purpose and found that OFP was effective at a lower temperature with no evidence of cavitation damage, suggesting that OFP is a better gas for drug delivery due to a higher vaporization efficiency compared to DFB [333]. Airan et al. developed a noninvasive targeted transcranial neuromodulation system using a propofol-loaded PEG-6 NE. This NE was created from polyethylene glycol-b-polycaprolactone block copolymer matrix filled with liquid PFC for US responsiveness in addition to biocompatibility and biodegradability. The drug release was limited to the brain and no evidence of damage to the brain parenchyma or blood–brain barrier was observed, indicating a good temporal and spatial control of drug release with this system [360].

Besides drug delivery applications, the NE phase transition under US irradiation can facilitate energy deposition by HIFU to improve tumor ablation [337]. Zhang et al. demonstrated that NEs could enhance the HIFU-mediated thermal ablation of tumors and efficiently accelerate the formation of HIFU thermal lesions. Bubbles derived from NEs reduced the required acoustic intensity for lesion formation by 89% in gel phantoms [361]. Shin et al. showed that 19F MRI could be used to quantitatively track the US-mediated ablation with PFC NEs [362]. Nanoemulsions have also been used as sonodynamic agents [363]. Zhang et al. fabricated a sonosensitizer using an IR780 dye incorporated in core–shell NDs. The core was filled with PFP while liposomal IR780 was loaded in the shell, and the sytem was tested for mitochondrial-targeted anticancer sonodynamic therapy. The results showed an increase in ROS generation in mitochondria which leads to cell death under US exposure. The US-mediated ADV process and the presence of IR780 facilitated the penetration and diffusion of the particles deep within the tumor, and the ability of IR780 for mitochondrial targeting was also verified. Moreover, under US irradiation IR780 showed concentration-dependent cytotoxicity. Another advantage of this system was the ability to be monitored and guided via multimodal (US, photoacoustic, fluorescent) imaging based on the ADV process [364]. Combinations of US with phase-transition NEs have also been investigated for thrombus detection and thrombolysis purposes [365]. In one interesting study, Guo et al. demonstrated a synergistic effect between US irradiation and NEs containing thyme essential oil to produce antimicrobial activity against *Escherichia coli* O157:H7 via alterations in cell morphology and damaging of internal structures. They hypothesized that US irradiation disturbed the bacterial membrane and cell wall integrity through a sonoporation effect and facilitated the penetration of NEs into bacterial cells [366].

Nanoemulsions can be hybridized with other materials in order to improve therapeutic or imaging applications. Fernandes et al. synthesized PFH NEs coated with silica–gold NPs for drug delivery and as multifunctional agents for photoacoustic (PA), US, and fluorescence imaging. It has been reported that silica-coated AuNPs could reduce the vaporization threshold. Moreover, the silica layer can transfer the heat more uniformly to the NEs, protect the AuNPs from melting under US irradiation, and enable drugs to be loaded onto the surface [367]. Gao et al. developed a new system based on Au nanorod (AuNR) hybrid NDs for synergistic photothermal and US-mediated gene delivery and simultaneous imaging. The NEs were fabricated from cationic poly(aspartamide) polymer and filled with fluorinated PHP. They were then attached to AuNRs and loaded with plasmid DNA. The AuNRs induced hyperthermia when irradiated with a near-infrared (NIR) laser, which promoted phase transition of PHP, while the US irradiation produced strong acoustic cavitation and sonoporation. As a result of this synergistic combination of NIR and US, contrast enhancement and gene transfection were potentiated [368]. Liu et al. hybridized NEs with superparamagnetic Fe_3_O_4_ and added folate as a targeting ligand for multimodal US, MR and PA imaging, and tumor destruction [345]. Nanoemulsions hybridized with liposomes have been explored for drug delivery applications and for contrast enhancement [339].

Nanoemulsions combined with US irradiation have been used to improve the efficacy of gene therapy. Gao et al. reported a 14-fold enhancement in the efficiency of gene transfer to HepE2 cells via phase transition of cationic droplets after US exposure at a frequency of 3.5 MHz [369]. In a similar study, they delivered a gene into Her2 positive cells. These particles also could act as US contrast agents [370]. In another study, Guo et al. fabricated US-responsive cationic nanodroplets filled with PFP and coated with poly(glutamic acid)-g-MeO-poly(ethylene glycol) (PGA-g-mPEG) as a shell in order to transfer the anti-proliferative miRNA-122 into hepatocellular cancer cells. Under US irradiation, these NDs undergo the ADV process and turn into MBs, which can penetrate the tumor cells and release the miRNA-122. The polymeric shell of the NDs also protected the miRNA-122 from degradation. Moreover, it was also shown that US irradiation did not cause any damage to the miRNA structure. The advantages of this system included a long blood circulation time, stability, biosafety, high gene transfer efficiency, and targeting ability [371]. Similarly, Dong et al. used NDs as carriers for pre-miRNA plasmids to treat hepatocellular carcinoma. They suggested that the sonoporation process was responsible for the transfection of the targeted cells [372].

An external stimulus, like a laser beam, can induce droplet-to-bubble transition, which leads to a significant increase in non-linear US signals [373]. It has been demonstrated that enhanced functionality can be achieved at a much lower pressure and laser intensity by the simultaneous utilization of US and laser through PA imaging [374].

#### Polymeric nanoparticles

Polymeric NPs include nanospheres, nanocapsules, and polymersomes [375]. The most widely utilized polymers are PLA, polycaprolactone (PCL), PLGA, and PS [376]. Polymeric nanoparticles can be fabricated via polymerization of monomers or by using preformed polymers. Synthesis methods can be categorized into one-step and two-step procedures. In one-step procedures, emulsification is not required for nanoparticle formation, whereas two-step procedures involve the preparation of an emulsification system followed by nanoparticle formation [377]. Since polymeric nanoparticles are made from natural polymers (e.g., chitosan, dextran, heparin, and hyaluronan) or biodegradable synthetic polymers (e.g., PLA, poly(glycolic acid) (PGA), and PLGA), they are considered biocompatible, biodegradable, and non-toxic [378].

**Figure 6:**
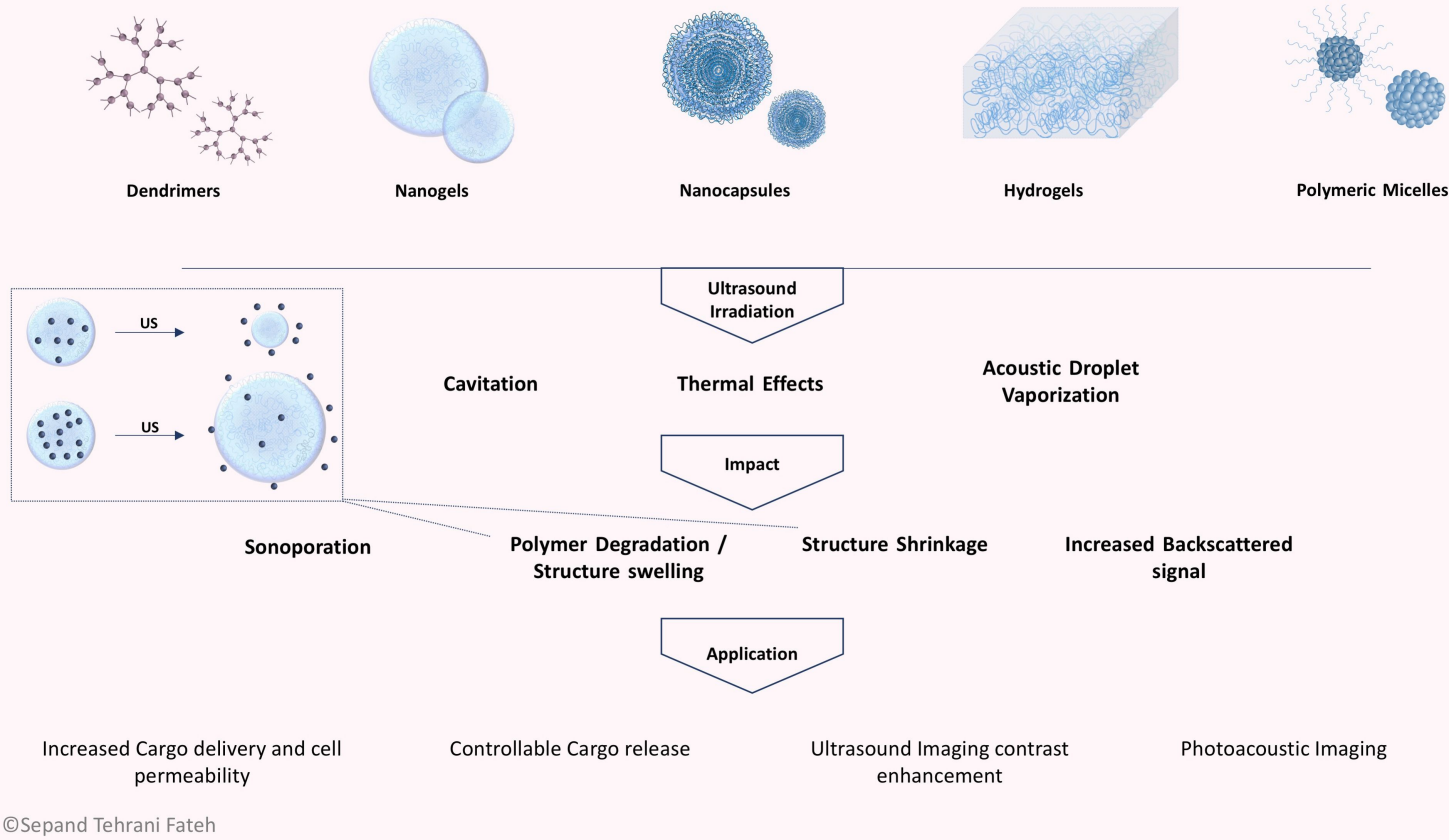
Mechanism of polymeric nanostructures in combination with US.

Ultrasound irradiation can degrade polymers through a mechanochemical process [379–380], in which the mechanical stress generates a chemical reaction. The mechanical stress is due to the rapid movement of the entangled polymeric chains which produce inertial cavitation shock waves and micro-streaming. Also, the possibility of cavitation-induced free radicals causing chain breakage might play a role in the polymer degradation process. Ultrasound irradiation could have an effect on solid polymers, as shown by Agrawal et al. [381], who studied solid copolymers of polylactic and polyglycolic acids submerged in phosphate-buffered saline. Although these polymers can naturally undergo hydrolysis in water, the rate of degradation with different molecular weight polymers was increased with the ultrasonic exposure time. Different aspects of the function and applications of polymeric nanoparticles are presented in Figure 6.

Polymers can be modified with various substances to improve their activity. For instance, polymeric NPs, which are copolymerized with PEG, can avoid recognition by mononuclear phagocytic cells [382]. Furthermore, the stability of polymeric NPs could be improved by the polymeric shell, and this would enable the NPs to survive in US pressure fields [383]. Nevertheless, numerous factors might influence the features of the nanocapsules, including an increased size [384]. Yang et al. prepared a new type of US-triggered biodegradable nanocapsules containing PFH, and the disulfide cross-linked poly(methylacrylic acid) (PMAA) shell was loaded with DOX [385]. The small PMAA–PFH nanocapsules were homogeneous (with a size of approximately 300 nm) and could accumulate in the tumor tissues via the EPR effect. In this system, DOX could be loaded up to 36 wt % in the PMAA shell and the drug loading efficiency was 93.5%. Under US irradiation, the drug could be rapidly released (<5 min). The PFH could improve the US imaging signal through the ADV effect. Additionally, the disulfide cross-linked PMAA shell was biodegradable and, consequently, non-toxic to biological organisms [386]. Solid polymeric NPs under US irradiation could decrease the cavitation threshold in water, even without the presence of preformed gas bubbles [387]. For example, polystyrene NPs can decrease the threshold for US-induced cavitation in pure water from about 7.3 to *<*5 bar depending on the size and concentration employed [388]. According to this study, the threshold decreased with increasing particle concentration and particle size [388]. As a result, even in the absence of gas bubbles, there was sufficient cavitation to produce substantial biological effects. In another study, the effects of polystyrene NPs (100 and 280 nm in diameter and concentration up to 0.2% w/w) on the cavitation threshold in water at a frequency of 20 kHz were examined. This approach was tested in vivo to increase the efficacy of cancer chemotherapy. The experiments were carried out in athymic nude mice bearing human colon KM20 tumors, which are resistant to chemotherapy. The delivery of the chemotherapeutic drug 5-fluorouracil was achieved by injecting polystyrene NPs plus 20 kHz US irradiation [388]. The combination treatment decreased the tumor volume and caused complete tumor regression at optimal irradiation conditions. Biodegradable PLGA NPs can improve the delivery of drugs, proteins, peptides, or plasmid DNA due to their ability to preserve macromolecules from degradation in endolysosomes [389]. The nontoxicity of PLGA-based NPs in clinical applications has been well proven [390], and PEG-conjugated PLGA NPs are presently being investigated as delivery vehicles with reduced systemic clearance in comparison to similar NPs without PEG [391–392].

In 2019, Zhang et al. reported an US-triggered pH-sensitive PLGA-based DOX delivery system [393]*.* They showed decreased side effects, such as cardiotoxicity and myelosuppression, caused by DOX and also increased therapeutic effects of DOX by combining US with chemotherapy. In this experiment, PLGA DDS had a size of 650 nm with high drug loading (15.8 ± 2.3%) and pH-responsive release properties. The in vitro results showed that DOX/NaHCO_3_@PLGANPs triggered by US displayed higher cell uptake and more inhibition on MCF-7 cells than free DOX or other formulations. An in vivo animal study showed that the relative tumor volume (0.63) of S180-tumor-bearing mice treated with US-activated DOX/NaHCO_3_@PLGANPs was lower than the control (0.81), DOX@PLGANPs without US (1.00), or free DOX (1.12). The synergistic effects of US and PEG on the release of a model drug (methylene blue) from PLA matrices were reported in another study [394]. By changing the US parameters they found that the drug release was controlled through a diffusion process and showed a good fit with the Higuchi diffusion model. The release of the MB from the DDS could be improved by PEG because of its high hydrophilicity and rapid dissolution speed.

Polymersomes are another common type of polymeric nanocarriers, which have been explored for drug and gene delivery. These polymers possess a synthetic vesicle membrane composed of amphiphilic block copolymers with a similar structure to the lipid bilayers in the cell membrane [395]. Self-assembly in an aqueous solution is a characteristic property of polymersomes, and this feature has enabled them to extensively be applied as DDS [396]. These artificial vesicles have a large interior compartment, with the advantages of having stability, an adjustable membrane, and the ability to encapsulate both types of compounds, (i.e., hydrophilic and lipophilic molecules). Their high loading capacity and ability to accumulate substances due to the EPR effect have led to them being studied for the controlled release of anticancer drugs [397]. An innovative polymeric vesicle based on a PEO-b-P (DEA-stat-TMA) (PEO: poly(ethylene oxide), DEA: 2-(diethylamino)ethyl methacrylate, TMA: (2-tetrahydrofuranyloxy)ethyl methacrylate) block copolymer, which could be activated with both US irradiation and pH changes in vitro, was reported by Chen and Du [398]. This dual-responsive vesicle had no cytotoxicity up to 250 mg/mL and could efficiently encapsulate drugs and release them under US irradiation or at lower pH values [386]. Recently, a new US-responsive polymersome preparation was described by the group of Jianzhong Du, which allowed endosomal escape for efficient cancer therapy [399]. These polymersomes allowed increased intracellular drug accumulation and enhanced tumor destruction. After ultrasonication, the endosomal escape occurred due to the proton sponge effect, and the drug was released into the cell nucleus. In vivo studies showed that drug-loaded polymersomes plus sonication inhibited tumor growth in a mouse model (95% reduction in tumor mass).

#### Chitosan nanocapsules

Chitosan (CS) is composed of *N*-acetyl-ᴅ-glucosamine and ᴅ-glucosamine units, linked by (1-4)-β glycoside bonds [400]. Chitosan nanoparticles can be manufactured through the following methods: ionotropic gelation, microemulsion, emulsification solvent diffusion, polyelectrolyte complex, and reverse micellar method. Ionotropic gelation and polyelectrolyte complex are the most widely used methods [401]. The type of application and the desired size requirements determine the synthesis method [402].

Chitosan enhances both paracellular and transcellular transport of drugs. Moreover, CS can bind to negatively charged mucus through ionic/hydrogen bonding/hydrophobic interactions with the anionic components. Physical and chemical modifications of CS can alter the properties of the particles either by the physical mixing of the polymers, known as blending, or by manipulation and conjugation of the functional groups. These modifications lead to mechanical improvement, more efficient drug encapsulation, and can enable particle responsiveness to external stimuli or triggers [403]. The disruption of CS particles can be triggered by pH changes, temperature, ionic strength, magnetic fields, or US waves. This triggering function could be due to the protonation/deprotonation equilibrium of the amino groups or to a phase transition [404].

The release of the encapsulated cargo can be caused by polymer swelling or by polymer erosion and degradation, and the balance between these mechanisms depends on the nature of the cargo, particle properties, surrounding medium, and the triggering stimulus [403]. The solubility, diffusion coefficient, biodegradation, size of the polymer matrices, and drug loading capacity are other parameters involved in the drug release profile [405].

Chitosan is frequently used in the fabrication of US-responsive nanomaterials [406–407] due to its natural origin and good biocompatibility. Most of the synergistic applications of CS plus US have been used due to the presence of other components in the CS nanohybrids. For instance, gas-filled CS nanocapsules and CS NDs can be ruptured by US, as discussed before [408]. Many studies have used CS NPs as a container for PFC liquids to form gas bubbles after US irradiation or as capsules to contain nanobubbles. The US-mediated droplet-to-bubble conversion known as ADV is responsible for the US-triggered cargo release and contrast enhancement using CS NPs [409–412]. Zhou et al. synthesized US-responsive CS nanobubbles for DOX delivery. Ultrasonication increased the cargo release by 46.45%, while the release of the non-ultrasonicated group was only 9.3%. Moreover, the uptake rate of DOX by the cells was much higher with DOX-loaded CS nanobubbles than with free DOX [408].

Kariminia et al. fabricated pH-responsive hybrid CS-coated iron oxide NPs (CS-Fe_3_O_4_) for dual-stimulus enhanced drug delivery (pH and US). They showed that the cargo release increased from 50 to 92% with US exposure time increasing from 0 to 60 min. Ultrasound irradiation disrupted the compacted structure of the CS NPs, leading to water absorption and drug release. Cavitation may also be a possible explanation [413].

Feng et al. encapsulated DOX in a water-soluble polyurethane and CS composite membrane as an US-responsive carrier in order to minimize the side effects of the toxic anticancer drug and maximize the controlled delivery and release of the drug into the targeted area. The hydrophilicity of WPU and the bioactive amino groups of CS provided biodegradability, cytocompatibility, and a longer blood circulation time to their complex. The cross-linked matrix protected the loaded drug from spontaneous release. They showed that ultrasonication significantly accelerated drug release from the complex due to changes in the matrix and degradation of the loosened structure. The drug release was dependent on the US parameters, so the desired rate of DOX release could be user-defined. The cavitation process after ultrasonication could also break down the nanostructure and release the drug. This complex showed good antitumor activity with less damage to healthy cells in comparison with free DOX [414].

#### Dendrimers

Dendrimers are another type of nanocarrier that has been used for drug/gene delivery [415–416]. Dendrimers are hyper-branched nanoparticles composed of monomers which form a symmetrical branched architecture around a core. Dendrimers can be fabricated trough two methods, including divergent and convergent methods. In the divergent method, the synthesis process is started from a core and monomers begin to polymerize in an iterative manner, forming several branches around the core. In other words, the direction of polymerization is from the core at the center to the outside. On the other hand, in the convergent method, monomers form branches without any relation to the core and the direction of polymerization is from the surface to the center of the structure. The product of this process is single branches. In the final step, these branches can be located on a core to form a dendrimer. Each step of the radial growth, regardless of the synthetic method, is referred to as dendrimer generation. The molecular weight and number of surface termini of the dendrimers are associated with their generation. The toxicity of the dendrimers is dependent on their physicochemical properties, such as terminal groups and surface charge [417]. Dendrimers can be fabricated from various materials, such as poly(amidoamine) (PAMAM), poly(propylenimine) (PPI), and poly(ʟ-lysine) (PLL); however, PAMAM dendrimers are the most famous in the literature for this purpose [417]. PAMAM dendrimers containing repeating ethylenediamine units in a tree-like expanding starburst structure have been investigated for biomedical applications [418–419]. PAMAM dendrimers are cationic nanostructures produced by the stepwise addition of spherical layers of methyl acrylate, followed by EDA, starting from one core molecule of EDA. PAMAM dendrimers possess exceptional molecular features, including welgos and an l-defined structure, a highly branched spherical structure, and low polydispersity [420–421].

Dendrimers can be divided into three regions: central core, space between branches, and their surface. Based on the physicochemical properties of the building blocks of the dendrimers, different cargo with hydrophobic or hydrophilic nature can be loaded in these regions. The surface of the dendrimers can also be functionalized for multifunctional or targeting properties [417,422]. Exceptional features of dendrimers can make them a suitable candidate for cargo delivery applications. Dendrimers can form non-covalent bonds with negatively charged drugs, nucleic acids, or plasmids and can be covalently conjugated with other cargos or ligands [423]. These nanomaterials have been injected intravenously for tumor targeting [424–425], administered orally for opening the tight junctions of epithelial barriers [426–427], and applied topically for drug delivery through the skin [428].

Dendrimers have been investigated for US-mediated transdermal drug delivery using acoustic cavitation to create small pores in the stratum corneum by temporarily disrupting the lipid bilayers [419,429–431]. Huang et al. demonstrated that the transdermal delivery of PAMAM dendrimers and their penetration through the skin can be improved via ultrasonication. Sonoporation is recognized as the underlying mechanism of this process [432]. Similarly, Manikkath et al. combined PAMAM dendrimers with low-frequency US for transdermal delivery of ketoprofen. Both PAMAM dendrimers and sonophoresis could each individually improve the transdermal penetration to some extent; however, the synergistic combination of dendrimers and US would lead to significantly higher drug penetration [419]. Other studies have shown that the use of US is beneficial to chemotherapeutic drug delivery, with inhibition of tumor growth and complete eradication in some cases [433–434]. In addition, US has been used to facilitate the regeneration of healthy tissue and hasten wound healing [435]. Different aspects of dendrimers as polymeric nanoparticles are shown in Figure 6.

#### Hydrogels

As a new generation of nanomaterials, hydrogels with three-dimensional networks in which water can be absorbed in comparatively high quantities play an imperative role in medical rehabilitation [436–437]. For applying hydrogels as DDS in the human body, an incredible biocompatibility is needed, which could be obtained by using biomass polymers, such as cellulose, chitin, and CS, and other polysaccharides whick keep large amounts of water [438–439]. Hydrogels obtained from biomass polymers for tissue engineering can be formulated through chemical cross-linking [440] and physical effects can be achieved through freezing-thawing [441] and phase-inversion processing [442]. Lately, phase-inversion hydrogels of cellulose have presented exceptional biocompatibility [443] and less cytotoxicity [444]. Such cellulose-based hydrogels loaded with drugs have been utilized as an US trigger for drug release [445]. The degree of cross-linking for a hydrogel that behaves as a sensor and actuator should be low enough to enable the polymeric network to experience remarkable conformational alterations in response to a specific stimulus, but high enough to offer the network mechanical stability to be able to preserve its functionality after several cycles of stimuli exposure. As a general rule, the rate of drug release from a chemically cross-linked hydrogel is affected by the size of the mesh (i.e., the free space between neighboring chains) which governs the drug diffusion rate. Rapid and adjustable modification of the degree of swelling in hydrogels in response to external stimuli or specific changes in the biological environment can be provided by introducing sufficient functional groups to alter the porosity and morphology of the structure [446]. Generally, a hydrogel will release the drug when it becomes swollen; however, the release rate decreases or even halts when it shrinks. However, the opposite behavior is occasionally observed since strong drug–hydrogel interactions can inhibit the release. In this case, when the hydrogel shrinks, the drug is released along with the ejected water [447]. *Mimosa pudica* root extract is an intriguing natural product which has been used to treat wounded skin. This preparation was loaded into cellulose hydrogel films and activated by US exposure, which cleaved the hydrogen bonds and released the drug [445]. Different aspects of the mechanism of action of hydrogels and their applications are summarized in Figure 6.

The release of PEGylated gold NPs from ionic cross-linked alginate hydrogels showed a dramatic increase in the in vitro release rate in response to US [448]. Bone morphogenetic protein-2 (BMP-2) was selected as a therapeutic agent to be linked to gold NPs, and could be released from the hydrogels by US irradiation. The BMP-2 maintained its bioactivity following alginate encapsulation and US triggered release. The non-encapsulated particles did not show any changes in the hydrodynamic radius or zeta potential after exposure to US waves. BMP-2–AuNPs were added to alginate microparticles and subsequently stimulated with US, and the resulting supernatant was added to mouse mesenchymal stem cells (D1 cells), leading to a two-fold increase in alkaline phosphatase (ALP) activity compared to osteogenic media control. The results showed that the NPs were physically trapped in alginate with only a low basal release rate that could be dramatically increased when triggered by US. This system had the potential to provide on-demand release by US irradiation and could be repeated over multiple days.

In another study [449], US-stimulated drug release was examined from a chitin hydrogel matrix loaded with gallic acid (GA), a drug that has been used for wound healing and has additional anticancer activity. The GA release from the GA–chitin hydrogel was examined under different US irradiation power values in the range of 0–30 W at 43 kHz. The results showed that US could accelerate the release in all the samples, and higher US power values, higher GA loading, and lower chitin concentrations were associated with a greater release of GA. The highest release rate of 0.74 µg/mL·min (nine times higher than that without US irradiation) was achieved with a GA concentration of 0.54 mg/cm^3^ and a 0.1 wt % chitin concentration under 43 kHz US irradiation at 30 W. It has been revealed that US irradiation made the material more rigid, with the possibility to break the hydrogen bonds in the GA–chitin hydrogels by measuring the hydrogel viscoelasticity and FTIR.

In 2017, Young and coworkers [450] studied the release profile of an *N*-isopropyl acrylamide-based hydrogel for US-triggered release of two large molecules, BSA (66 kDa) and dextran (3–5 kDa). The US waves could increase the release of BSA, and the mechanism was due to both thermal and non-thermal effects. Aside from heating, US increased the release of BSA much more than a simple water bath. The further increase in BSA release triggered by US was ascribed to the streaming effect caused by the propagation of US waves “pushing” the BSA molecules out of the hydrogel. In this study, a positive correlation between US intensity and BSA release rate was shown.

In another study [451], researchers developed an US-responsive material for the controlled release of a fluorescein-labeled transferrin conjugate and a fluorescein–lysozyme (from hen egg white) conjugate regardless of their electrical charge and structure. The supramolecular polymeric hydrogel was cross-linked with a host–guest interaction of β-cyclodextrin and adamantane in order to enclose two types of model proteins and could site-specifically release the protein cargos in a stepwise manner after US activation without losing their activity. Protein delivery to living cells via US has been demonstrated on model tissue comprising cells plus extracellular matrix. This study showed that the supramolecular polymeric hydrogel had the potential to be used as a carrier in an US-guided protein delivery system.

An injectable, biocompatible, and thermosensitive hydrogel system, mPEG-PLGA-BOX (BOX = 2,2'-bis(2-oxazoline)) block copolymer for US-triggered drug release was reported [452]. The viscosity of a 15 wt % hydrogel was 0.03 Pa·s at 25 °C (liquid form) and 34.37 Pa·s at 37 °C (gel form). The baseline and US-activated in vitro release profiles of a small molecule drug (DOX) and a large molecule (FITC–dextran) were measured. A long-lasting baseline release rate was measured in vitro over seven days. When the DDS was triggered by US (1 MHz, CW, 0.4 W/cm^2^), the release rate increased nearly 70 times. When the US was switched off, the release rate returned to baseline. The in vivo release profile of DOX was measured after subcutaneous injection into the back of mice and rats. The results have shown that the hydrogels remained in situ and provided a steady release for at least seven days. After US application, the in vivo release from the hydrogel was increased by ≈10-fold. Thermal effects were suggested as the proposed mechanism because the temperature was raised to ≈40 °C in vivo after exposure to US (0.4 W/cm^2^). The blood concentration of DOX after US treatment was measured. There were no statistically significant differences in blood DOX concentration with and without US irradiation. Consequently, local release to the surrounding muscle was demonstrated, which confirmed localized US-responsive drug release. The increased concentration of DOX in muscle but not in the blood was explained by the fact that the increased concentration in the blood could be too small compared to the baseline to be detected; therefore, systemic toxicity was unlikely.

A novel US-responsive transdermal DDS was reported by Huang et al. [453], who embedded diclofenac sodium (DS) into four-armed PEG–polyester microcapsules inside a hydrogel patch. They assessed the in vitro release profile and drug release after US irradiation at 37 °C with or without continuous or intermittent US exposure. In the presence of US, the release of DS reached 90% at 8 min, while without US only <20% of the DS was released. To further assess the effectiveness of the (DS@PEG-PLGA)@PEG hydrogel patch as a transdermal DDS, in vivo experiments were done in a rat model. The hydrogel patch was adherent to a shaved area of the rat abdomen and the drug release with and without US was analyzed. With the assistance of US, the DS was released and rapidly penetrated into the subcutaneous tissue in a time-dependent manner. Without US, only negligible DS was detected after 6 min. The small amount of drug release may be due to simple diffusion and the intrinsic permeability of rat skin.

#### Nanogels

Nanogels are a colloidal dispersion of hydrogel NPs produced from physically or chemically cross-linked polymeric networks [454]. Nanogels are hydrogels with a nanoscale size and can overcome some of the limitations of macrosized hydrogels [455]. Nanogel networks are often composed of synthetic polymers, such as PLA, PCL, PLGA, polyacrylates, or polymethacrylates. They can also be produced from natural polymers, including proteins (collagen, gelatin, albumin, or fibrin) or polysaccharides (CS, hyaluronic acid, heparin, agarose, or alginic acid) [456]. Cellulose, chondroitin sulfate, pectin, dextran, cyclodextrin, and pullulans are some other components of nanogels [457]; however, polysaccharide nanogels are overall considered to be harder to fabricate [457].

The advantages of nanogels include high water content, large surface area, good stability, bioconjugation of active targeting agents, biocompatibility, prolonged circulation time, high loading capacity, ease of preparation, low toxicity, and flexibility in their design [455–457]. Moreover, nanogels can facilitate the cellular internalization of drug cargos. Nanogels with a size in the range of 20–359 nm have all shown more or less internalization by different types of cells [458]. Despite the good biocompatibility of proteins assembled into a nanogel network, the uncontrolled structure, degradability, poor drug release, and their potential to induce immunological reactions have limited their in vivo applications. In contrast, synthetic polymers possess well-controlled structures and biodegradability, good stability, and can carry out targeted drug release. The lack of intrinsic biological activity is one limitation of synthetic polymer-based nanogels [459]. A combination of natural and synthetic polymers could be a possible way to develop nanogels with improved biological properties [459]

Routes for nanogel preparation can be divided into physical cross-linking or chemical cross-linking. Physical cross-linking is based on hydrogen bonds, van der Waals bonds, and electrostatic interactions. Chemical cross-linking is based on the formation of covalent bonds between functional groups present on the polymer chain [455]. Nanogel preparation methods include electrostatic interactions, reverse miniemulsion, desolvation/coacervation, hydrophobic interactions, and cross-linking of micelles [459]. Some parameters, including size, shape, surface chemistry, and charge should be taken into account for efficient drug delivery [456].

Nanogels have been widely explored as cargo carriers. The release of cargo from nanogels can be triggered with different stimuli, including redox potential [460–461], pH changes [462–463], salt concentration [464], US [29], temperature [465], or light [466–468]. Also, it is possible to render nanogels responsive to specific stimuli, such as magnetic fields [469], by decorating nanogels with magnetic NPs. The stimulus-responsive properties are due to the cross-links between the nanogel chains and branches which become unstable when subjected to certain stimuli, leading to disruption, degradation, and triggered release of the cargo [470]. Recent studies about stimulus-responsive nanogels have been reviewed in [471–473]. Ultrasound irradiation can induce cargo release due to the increased permeability of the nanogel network, likely because of cavitation processes [474].

Di et al. coated insulin-loaded PLGA nanocapsules with microgels in order to prepare an US-triggered structure capable of pulsatile insulin release. Insulin passively diffused from the nanocapsules into the microgels. After US exposure, the insulin in the microgels was released in a rapid burst in addition to a long-lasting sustained release. The acoustic peak pressure, pulse duration, and the duration of US application affected the release efficacy. The authors proposed that the mechanism could be explained by a cavitation process because significantly less insulin was released in degassed PBS solution compared to control samples [475].

In another study, a urokinase-type plasminogen activator (UPA) was encapsulated into hollow nanogels for selective thrombolysis. Ultrasound exposure at 2 MHz triggered the release of 90% of the UPA within 1 h, leading to enhanced clot thrombolysis. They suggested that the cargo release was due to an US-mediated deformation of the relatively soft hydrated nanogels. Nanogels loaded with UPA plus US showed similar thrombolytic activity compared to pure UPA, confirming that the bioactivity of UPA was fully preserved [476]. In a similar study, the authors evaluated the sonothrombolysis activity of UPA-loaded nanogels. The enhanced UPA activity and prolonged UPA circulation time enabled better protection for the blood–brain barrier compared to free UPA [477].

Heo et al. fabricated a peroxamide-based US contrast agent capable of generating CO_2_ in the presence of H_2_O_2_ and US for imaging inflammatory diseases. Highly concentrated peroxamide and a basic catalyst served as a reactor for a chemiluminescence reaction, which was responsible for CO_2_ gas generation leading to US contrast enhancement. The contrast enhancement was attributed to the H_2_O_2_-responsive bubbles while the H_2_O_2_ concentration was high. This feature of the fabricated nanogels makes inflammatory tissue imaging possible [478].

Other researchers have used PFH encapsulated within nanogels as a phase transition agent for US-responsive drug delivery and imaging. Ultrasound irradiation caused vaporization of PFH, which led to cargo release and contrast enhancement. Ultrasonication may also affect the structure of the nanogel independently of the vaporization process. The combination of this US-responsive complex with a reducing agent provided 90% of drug release within 10 min [29].

Nanogels can also be used as contrast agents and as cargo carriers at the same time. Wang et al. evaluated dual-enzyme-loaded (catalase and superoxide dismutase) multifunctional glycol CS nanogels as probes for dual-modality US imaging and T2-weighted MRI [479]. The results showed that the NG probes interacted with pathological reactive oxygen species to enhance the concentration of molecular oxygen in an acidic environment for enhanced US imaging and T2-weighted MRI. Chen et al. fabricated a novel stimulus-responsive nanogel-based contrast agent for PA imaging in order to increase the signal up to 30 times and eliminate the intrinsic background noise leading to a 5-fold enhancement in in vivo contrast. They loaded PA contrast agents, such as gold nanorods or copper sulfide nanospheres into a poly(*N*-isopropylacrylamide) nanogel as a volume-changing photothermal stimulus-responsive cargo carrier. By increasing the temperature above the “lower critical solution temperature”, the nanogel composite underwent shrinkage and its optical properties changed, leading to higher PA signals. The shrinkage of the structures and their controlled aggregation in response to the raised temperature reoriented the gold nanorods to bring them closer to each other, resulting in a shift of the plasmonic resonance wavelength [480]. Different aspects of the functions and applications of nanogels are summarized in Figure 6.

#### Gold nanoparticles

Gold NPs (AuNPs) have attracted tremendous interest due to their chemical and physical properties, which makes them a suitable candidate for many therapeutic and diagnostic applications, such as drug delivery, PA contrast enhancement, biological labels, biosensors, catalysts, photodynamic therapy, photothermal therapy, and X-ray imaging contrast agents [481]. The first scientific report describing the production of AuNPs dates from 1857. However, reports dating from the 5th and 4th centuries BC in Egypt and China suggested the use of soluble gold for anesthetic and curative purposes. Until the middle ages, soluble gold was used to treat and diagnose a range of diseases [482]. Some recent biomedical applications of AuNPs have been reviewed in [481–484]. Gold NPs can be synthesized either by physical methods (UV radiation, sonochemical, microwave radiation) or by photochemical, chemical, and biological procedures [481]. Despite the general opinion that AuNPs are non-toxic materials, there is still no absolute certainty of this fact [485]. Several variables, such as particle size, shape, surface chemistry, dosage, time of exposure, route of administration, and cell type may all be involved in the possible toxicity of AuNPs [486].

**Figure 7:**
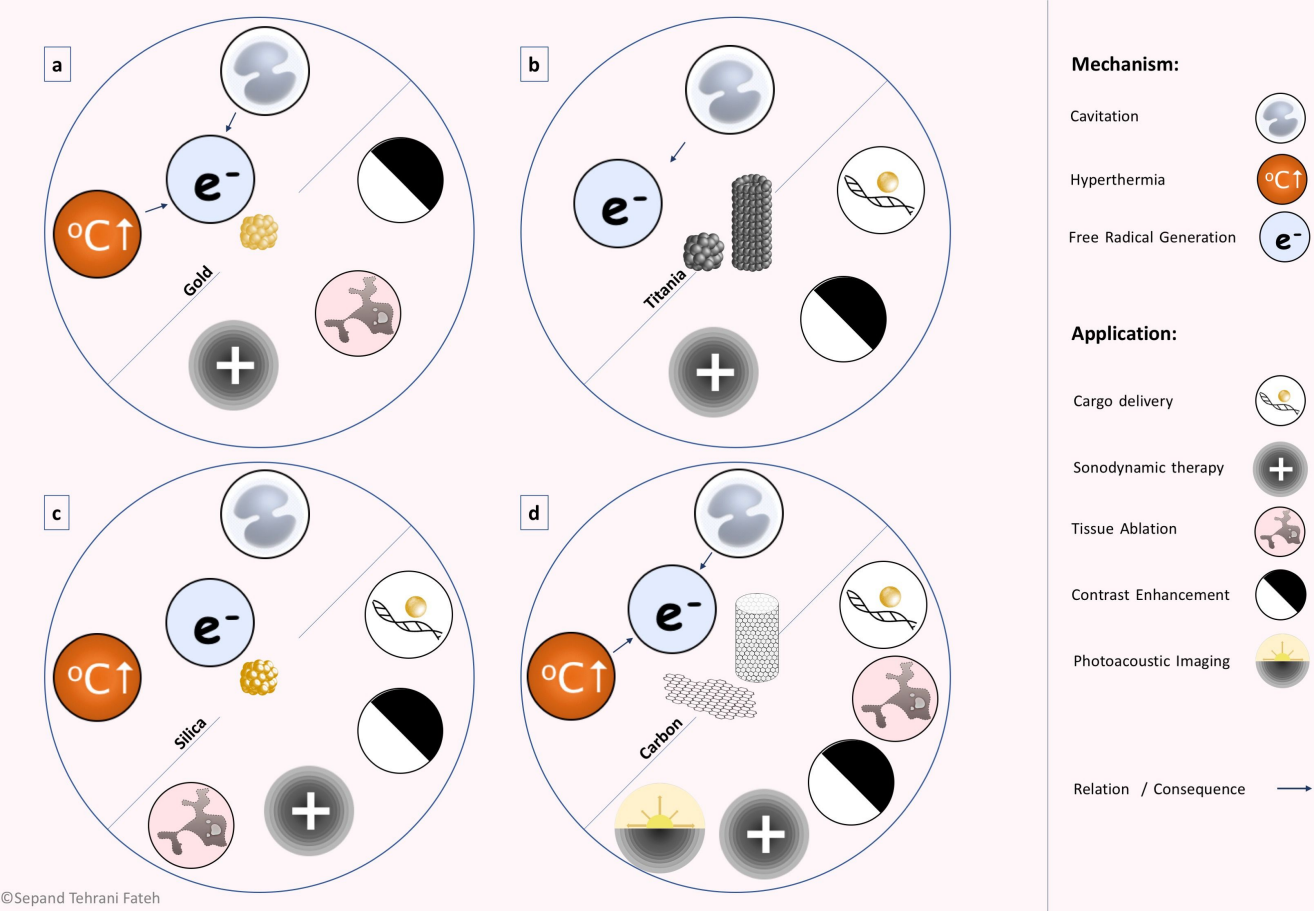
Mechanisms of action of (a) gold, (b) titania, (c) silica, and (d) carbon nanostructures plus US irradiation. Hyperthermia, cavitation, and free radical species generation can occur alone or in combination.

The mechanism of action of the therapeutic and diagnostic applications of AuNPs in combination with US irradiation can be divided into four possible pathways [487]: AuNPs could increase the attenuation coefficient of the US waves in the medium [488], AuNPs could absorb energy leading to hyperthermia [489], AuNPs could act as a nucleation point for cavitation bubble formation and decrease the cavitation threshold [490], and AuNPs could generate free radicals and ROS [491] (Figure 7).

De Oliveira Gonçalves et al. produced AuNPs loaded with aminolevulinic acid (ALA-AuNPs) to act as a sonosensitizer for sonodynamic therapy in atherosclerosis. They detected the generation of singlet oxygen during US irradiation, which reduced the macrophage viability in the atherosclerotic plaques [491]. Another group of researchers produced AuNPs with attached folate residues for targeted sonodynamic therapy of cancer cells. A 90% reduction was observed only in the folate receptor-expressing cancer cells after 72 h, confirming that AuNPs could function as a targeted sonosensitizer [492]. Similarly, Deepagan et al. developed an Au–TiO_2_ nanocomposite to enhance sonodynamic therapy of tumors. In this study, the hydrophilic Au–TiO_2_ nanocomposite and hydrophilic TiO_2_ NPs were compared to each other. Despite their similar physicochemical properties, the Au–TiO_2_ nanocomposite generated more ROS with US, and produced a 3.11-fold tumor shrinkage in comparison to TiO_2_ NPs. This was suggested to be because the AuNPs absorbed more energy and caused hyperthermia [493].

Beik et al. injected AuNPs into BALB/C mice bearing CT26 colorectal tumors and irradiated the targeted area with US producing tumor shrinkage without any relapse. It was concluded that the sonosensitizing properties of AuNPs were due to thermal and mechanical effects of ultrasonication [494]. Similarly, Devarakonda et al. also studied hyperthermia with AuNPs under US irradiation [489]. Shanei et al. demonstrated that AuNPs played a role in the cavitation nucleation process and could decrease the cavitation threshold. According to their study, the number of cavitation bubbles was higher with increased particle size due to the increased number of nucleation sites [490]. McLaughlan et al. reported similar results and stated that AuNP-mediated cavitation bubbles could improve HIFU therapy [495].

Coluccia et al. tested cisplatin-conjugated AuNPs in combination with MR-guided FUS for glioblastoma treatment. The results showed tumor growth inhibition, DNA damage, and more cell death compared to free cisplatin [496]. Sun et al. prepared contrast agents for US imaging based on gas-generating AuNPs. They used AuNPs as a photocatalyst and modified the surface with 4-azidobenzoic acid groups as a gas precursor by conjugation to the amine groups of glycol–CS-coated AuNPs. The use of AuNPs as a photocatalyst enabled the use of longer electromagnetic waves, such as visible NIR rather than UV. Visible and NIR light can penetrate deeper into the tissue to activate AuNPs. Under laser irradiation, AuNPs catalyzed photolysis of the azide groups on their surface and N_2_ MBs were formed. These MBs could enhance the contrast of US imaging due to backscattered US signals. These nanostructures were smaller than MBs; therefore, they showed better penetration into the target tissue. Moreover, their small size and negative zeta potential after gas generation led to a longer blood residency time and better clearance. This system was controllable, had broad spectrum responsiveness, and was able to be used in other imaging modalities [497].

#### Titania nanostructures

Titania (TiO_2_) nanostructures can act as cargo carriers with various forms and compositions [498]. TiO_2_ nanostructures can take the shape of nanorods, nanowires, nanosheets, nanofibers, or nanotubes [499]. Sol–gel, hydrothermal, solvothermal, or electrochemical anodization are some of the conventional methods for the fabrication of titania nanostructures, which have been reviewed in [498]. Low toxicity, good biocompatibility, good stability, intrinsic properties, and versatile fabrication techniques are some of the advantages of TiO_2_ nanostructures [500]. However, they can potentially affect protein conformation and induce ROS generation [500]. The biomedical applications of titania nanostructures include tissue engineering, drug delivery, biosensors, sonodynamic therapy, and antibacterial activity [501–503]. These structures can be functionalized with different biomolecules for biomedical applications, as reviewed in [504]. Titania nanostructures have been used in stimulus-responsive systems triggered by pH [505–506], magnetic or electric fields [507], light [508], NIR [500], and US [509] either alone or as hybrids with other nanostructures.

Titania nanostructures have been extensively used in sonodynamic therapy either alone or hybridized with other nanostructures [510–512] (Figure 7). TiO_2_ NPs can also enhance the sonosensitizing activity of sonosensitizers [513]. Shimizu et al. reported that the presence of TiO_2_ during ultrasonication accelerated the generation of hydroxyl radicals. They proposed that this phenomenon might be due to cavitation, in which TiO_2_ acted as a primary nucleus for this process by creating thermally excited positive holes [509]. The same group of scientists tested TiO_2_ NPs for killing cancer cells and investigated the uptake process. Moreover, they demonstrated that a synergistic combination of targeted TiO_2_ NPs plus ultrasonication caused apoptosis as a result of radical generation and physical stress [514]. They also modified TiO_2_ NPs with avidin in order to target breast cancer cells. After 1h of incubation 30% of the normal cells and 80% of the cancer cells had taken up particles. They demonstrated that the uptake of these particles alone (without US) did not show toxicity. They also showed that TiO_2_ NPs could act as nuclei for the cavitation process, and OH radicals were generated due to the thermal excitation of TiO_2_ NPs [515]. In 2016, these scientists investigated Ni–TiO_2_ alloy as an OH radical-generating sonocatalyst with a 50% reduction in viability of cultured MCF-7 cells after ultrasonication [516].

Shi et al. developed a novel drug carrier based on mesoporous TiO_2_ NPs. These NPs were loaded with docetaxel, and β-cyclodextrin was attached to the surface of the NPs to act as a gatekeeper to control drug release using a ROS-sensitive linker. Ultrasound irradiation produced ROS, which led to the cleavage of the ROS-sensitive linker and detachment of the β-cyclodextrin allowing rapid cargo release. This structure could also be used as a sonosensitizer for sonodynamic therapy since it is a ROS generator. In vitro and in vivo studies showed decreased cell viability and tumor shrinkage, respectively [517]. In a similar research, Kim et al. [518] fabricated a TiO_2_ NPs-based platform for controlled delivery of DOX which works based on sonosensitizing properties of TiO_2_ NPs. They encapsulated DOX-coordinated TiO_2_ NPs with polymeric phenylboronic acid (pPBA) via an ester bond between pPBA and DOX. The phenylboronic ester bond can be cleaved subsequently to an exposure to ROS generated from TiO_2_ NPs after US irradiation. The generation of ROS also facilitates the anticancer function of this system.

You et al. also fabricated TiO_2_ NPs for sonodynamic therapy. Their hydrophilized TiO_2_ NPs caused an elevation in pro-inflammatory cytokines and a 15-fold decrease in the size of the tumor after ultrasonication. They used a fluorescent probe-based technique to detect the presence of ^1^O_2_ in the treated tissue, which was 29.7-fold higher in comparison with no US-treated samples [154].

Another team developed triple multifunctional TiO_2_–Gd NPs as drug carriers, sonosensitizers, and MRI contrast agents. The presence of Gd atoms not only enhanced the MRI contrast but also improved the sonodynamic activity of TiO_2_. The synergism of this complex with US produced up to 91.68% cell death through US-mediated ROS generation. In vivo and in vitro studies confirmed the effectiveness of US-triggered chemo–sonodynamic therapy and high-quality MRI images [519]. Liang et al. fabricated Pt–TiO_2_ nanostructures in order to enhance sonodynamic therapy by overcoming two problems of this technology: the low quantum yield of current sonosensitizers and the tumor hypoxic environment. Titania NPs decorated with Pt NPs provided an oxygen-deficient layer that could act as a nanoenzyme based on their antioxidant activity. Under US irradiation, TiO_2_ NPs produce singlet oxygen, OH, and superoxide radicals which can activate apoptosis, while simultaneously, Pt NPs converted H_2_O_2_ into free O_2_ molecules which could improve the oxygenation of the hypoxic tumor environment. The hollow TiO_2_ NPs also acted as a reservoir for DOX, which is not only a chemotherapy drug but could also act as an additional sonosensitizer for ROS generation [511]. Zhang et al. described titania-coated Au nanoplates for synergistic photothermal/sonodynamic therapy. The generation of ROS from this complex was increased due to the ability of Au to entrap electrons in comparison with pure TiO_2_, resulting in cell apoptosis [512]. Similarly, Cao et al. synthesized TiO_2_ nanosheets with Au clusters attached to their edges to act as sonosensitizers. These composites were also modified for mitochondrial targeting by attaching triphenylphosphine, and were loaded with the AS1411 aptamer. The results showed an improvement in the quantum yield of Au–TiO_2_ nanosheets in comparison with pure TiO_2_ and Au–TiO_2_ NPs. It was shown that the nanostructures were taken up by the cancer cells via endocytosis and then escaped from lysosomes and accumulated in the mitochondria leading to apoptosis and necrosis. They also stated that they showed minimal toxicity and no side effects. According to their study, Au–TiO_2_ nanostructures exhibited good biocompatibility, hydrophilicity, and had a long circulation time. They could also be used as computed tomography (CT) imaging contrast agents due to the high atomic number of Au [520].

Gao et al. fabricated needle-like TiO_2_ NPs in the form of polyelectrolyte capsules via the hydrolysis of titanium butoxide, precipitation, and layer-by-layer assembly, which led to UV–US dual-responsive microcapsules. Ultraviolet or ultrasound irradiation could trigger irreversible shell rupture and efficient cargo release. They also confirmed the biocompatibility of these particles using a 3‐(4,5‐dimethylthiazol‐2‐yl)‐2,5‐diphenyltetrazolium bromide (MTT) assay. They suggested that the increased density gradient across the water/shell interface, the enhanced shell stiffness, and the decreased shell elasticity might be some of the reasons for the increased US responsiveness due to a better absorption of acoustic energy [521]. Wang et al. used ultrafine titanium monoxide nanorods and suggested that these structures were more efficient than TiO_2_ NPs in ROS generation for sonodynamic therapy [522].

Zhou et al. fabricated superhydrophobic TiO_2_ nanotubes with a trapped air layer on their surface. The superhydrophobicity prevented undesired cargo leakage and allowed efficient drug loading. After exposure to US, the air layer was removed and the cargo was subsequently released [523] (Figure 7).

#### Carbon nanostructures

There are many carbon nanostructures including fullerenes, carbon nanotubes (CNTs), graphene, and carbon quantum dots, which all contain sp^2^-bonded carbon atoms. The electrical, chemical, and mechanical properties of these structures make them attractive for diverse biomedical applications [524]. Carbon nanotubes were first reported in 1991 and since then many studies have been carried out in order to characterize and utilize these structures [525–526]. Carbon nanotubes can be thought of as graphene sheets rolled into a seamless cylinder, and they can be either open-ended or capped [526]. They can be found either as single or concentric multilayered nanotubes [524]. Plasma-based and thermal synthesis methods are most common procedures for CNT fabrication [527]. Carbon nanotubes have low solubility in aqueous solution, whereas functionalization can lead to better solubility and the possibility of cargo loading [528]. Carbon nanotubes have been extensively used in biomedical applications, such as biosensors, cargo carriers, PA imaging agents, cancer therapy, implants, photothermal therapy, tissue engineering, and regenerative medicine [524,529–531]. Up to now, despite many investigations, there is no consensus about the biocompatibility and safety of CNTs, but it has been suggested there might be a close relationship between parameters, such as surface functionalization and concentration of the CNTs and their toxicity [526]. Number of walls, surface area and chemistry, shape, size, length, functionalization, and defects are some of the factors affecting the toxicity of CNTs [531]. Carbon nanotubes can cause toxicity through oxidative stress, membrane injury, genotoxicity, and interactions with the immune system [532]. Carbon nanostructures could be advantageously combined with metal NPs for more novel and multifunctional applications in diagnosis and treatment due to the synergism between the properties of both particles [533]. Carbon nanotubes have been used with US irradiation for therapeutic and diagnostic applications (Figure 7).

Delogu et al. fabricated functionalized multiwalled carbon nanotubes (MWCNT) with a diameter of 20–30 nm and a length of about 400 nm as US contrast agents. They oxidized and then functionalized the CNTs by 1,3-dipolar cycloaddition of azomethine ylides in order to provide biocompatibility. Their results demonstrated long-lasting US contrast properties. Moreover, the US signal of functionalized multiwalled CNTs was higher than that of graphene oxide, pristine multiwalled CNTs, or single-walled CNTs and was equal to that of sulfur hexafluoride (a commercial contrast agent). It was reported that multiwalled NTs were highly echogenic in the liver and heart, and it was possible to visualize a pig bladder with low-frequency US. No toxicity was reported seven days after the injection [534]. Similarly, Gu et al. synthesized multiwalled CNTs modified with PEG and an anti-prostate-specific membrane antigen (PSMA) aptamer as a targeted US contrast agent. Their results suggested that CNT-based contrast agents were more accurate, with an enhanced image contrast compared to traditional contrast agents, and were capable of targeting the PSMA-expressing cells [535]. Ding et al. developed a multifunctional contrast agent for both microwave-induced thermoacoustic imaging and MRI based on CNTs. The authors incorporated ferromagnetic materials into multiwalled CNT in order to add electrical and magnetic properties to the complex. Their results showed that these complexes caused a 67% enhancement in thermoacoustic imaging and an 80% decrease in T2 signal intensity in comparison with tubes without ferromagnetic materials [536].

Wu et al. functionalized MWCNTs with polyethylenimine followed by conjugation to FITC and an anti-prostate stem cell antigen monoclonal antibody in order to enhance the signal in US imaging and allow targeted drug delivery. They demonstrated that this complex had good biocompatibility and was bound to and was taken up by target cells, enhancing the US signal intensity and contrast. The MWCNTs acted as the pivotal core of this contrast agent and cargo carrier, while the conjugated antibody played an important role in targeting. Hence, it could be potentially used in real-time tumor monitoring. The signal enhancement was observed even at 10 mg/mL concentration, much lower than that of hollow silica microspheres. Moreover, the complex accumulated in the tumor environment and released the loaded drug to inhibit the tumor growth, demonstrating its potential in theranostic applications [537]. Wang et al. used COOH-functionalized multiwalled CNTs as a carrier for protohemin to act as a sonosensitizer. They demonstrated that the protohemin-conjugated MWCNT–COOH showed a greater inhibitory effect under US exposure compared to protohemin alone [538]. He et al. developed PFH-encapsulated fullerenes as a multifunctional complex and as an US/CT contrast agent for HIFU therapy. The probable mechanisms of HIFU ablation were cavitation, sonochemical reactions, and thermal effects leading to ROS generation [539].

Other carbon nanostructures have also been used in synergistic combination with US for both diagnostic or therapeutic applications. Yu et al. developed graphene oxide conjugated with a pillar[6]arene-based host–guest complex (CP6
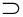
PyN) for US contrast enhancement and PA imaging. This structure was capable of generating CO_2_ nanobubbles under NIR light exposure, producing US contrast enhancement. Under NIR irradiation, the photothermal effects of graphene oxide caused CO_2_ generation from the bicarbonate counterions at the surface of the structure [540]. In another study, Pan et al. investigated metal–organic framework-derived mesoporous carbon nanostructures for sonodynamic therapy. These structures were composed of a zeolitic imidazolate framework coated with a mesoporous silica shell modified with PEGylated vitamin E and then subjected to a carbonization process. It was shown that hydroxyl radicals (•OH) and singlet oxygen (^1^O_2_) were generated under US irradiation leading to a tumor inhibition of 85%. High ROS generation was due to a large bandgap between the highest occupied molecular orbital and the lowest unoccupied molecular orbital of this complex. Moreover, this structure could also enhance MB formation by acting as a nucleation site leading to a decrease in the cavitation threshold intensity. High ROS generation, good stability, excellent gas adsorption, deep penetration into the tissue, suitable biodistribution, and biocompatibility were some of its advantages [541].

#### Silica nanostructures

Silica (SiO_2_) nanostructures have been extensively used for US-responsive drug delivery and US contrast enhancement. Among these nanostructures, most studies have been devoted to MSN due to their favorable properties, which will be discussed in the following section. MSNs display high thermal and chemical stability, resistance to corrosion under harsh conditions, unique optical properties, low density, and a high adsorption capacity for many cargo molecules. Large-scale synthesis of a range of different morphologies and textures is possible, and a high drug loading capacity is due to a high surface area and volume of the mesopores. The size of a mesopore is tunable and its surface can be functionalized to enable clearance and excretion from the body. Their physicochemical properties can be tailored to provide sensitivity to various stimuli, targeting ability, biocompatibility, biodegradability, and controlled release of encapsulated cargos [542–546]. The industrial large-scale preparation of MSNs is still under investigation, which might be a barrier on the way towards commercialization [547]. MSNs can be used in applications for drug delivery, diagnostic imaging, biocatalysis, biosensors, enzyme supports, protein adsorption and separation, and nucleic acid detection and purification [542,544]. MSNs are suitable platforms for multi-component drug delivery due to their high surface area and well-defined mesopores. Small molecules can be loaded into mesopores, while the outer hydrophilic surface allows for the loading of large biomolecules, such as proteins and nucleic acids [545]. MSNs have been used in the delivery of drugs, DNA, siRNA, growth factors, and enzymes [542,548–549]. These NPs can be combined with contrast agents and fluorescent reporters for diagnostic applications [550–554]. MSNs, their composites, and biomedical applications have been reviewed in [555–557]. MSNs can be modified to be responsive to multiple triggers and they possess high functionalization potential, which can potentially enhance both therapeutic and diagnostic efficacy [542]. MSN-based nanostructures can be triggered via NIR light [558], US [559], magnetic fields [560–561], electricity [562], or temperature [563] as examples of exogenous stimuli. Redox potential [564–565], pH changes [566], enzyme activity [567], glucose [568], and ATP [569] concentrations are examples of endogenous stimuli. Stimulus-responsive silica nanostructures have been reviewed in [570–572]. MSNs can be coated with polymers to act as pore gatekeepers, and these can be made responsive to the aforementioned stimuli [20].

As mentioned above, MSNs are ideal cargo carriers due to their advantageous properties. The intended drug is loaded within the pores and to prevent the pores from being prematurely opened, some sort of cap should be grafted onto the pores as a gatekeeper to limit unwanted cargo release [543–544]. These caps can be stimulus-responsive in order to achieve controlled or on-demand cargo release [547]. Two types of cargo can be loaded without any need for a cap: non-covalently loaded hydrophobic cargos and non-covalently loaded hydrophilic cargos [546]. Opsonization is among the most important problems that MSNs encounter during in vivo administration and PEGylation, zwitterions, or lipid coatings would prevent it. On the other hand, adjusting the electrostatic charge or active targeting could be used to enhance the cellular uptake and delivery of cargo [543]. Increasing the hydrodynamic diameter and changing the surface electrical charge are two ways to prevent protein corona formation in physiological environments. This phenomenon could have both positive and negative impacts on MSN suitability for biomedical applications [546]. MSNs can be functionalized with active ligands for cell targeting, such as folate for cancer cells and CD44 for increasing endocytosis [542].

Four general methods are available for MSN preparation, including template-directed methods, sol–gel methods, microwave-assisted methods, and chemical etching techniques [542,546]. Silica NPs can be fabricated with a core–shell formulation for theranostic applications with multifunctional properties, which have been reviewed in [573].

There is no accepted consensus about the cytotoxicity of MSNs and possible carcinogenesis [574–575]. Two possible mechanisms have been proposed for MSN cytotoxicity: the presence of surface silanol groups and ROS generation [574,576]. MSNs generally exhibit lower hemolytic effects than other silica NPs, which is due to fewer silanol groups being present in the mesoporous formulation [577]. However, the silanol functional groups can be easily modified with other functional ligands to improve MSN properties [542]. The biosafety of MSNs has been discussed in [574,578–579] and silica NPs in [580].

The biodistribution of MSNs is related to the preparation method, particle size, particle shape, surface chemistry, and administration route. Moreover, the cellular uptake is dependent on the dosage, cell type, and incubation time [574]. The size of the particles, surface functionalization, electrostatic charge, and morphology all affect the efficiency of MSN clearance [546]. Non-spherical structures, such as short and long rod-shaped MSNs, show different degrees of biocompatibility, biodistribution, and clearance in comparison to spherical NPs. In particular, rod-shaped MSNs show more cell internalization [581].

The synergistic use of silica NPs plus US irradiation has enabled various novel biomedical applications, including US imaging, sonodynamic therapy, HIFU tumor ablation, and US-triggered drug delivery [582] (Figure 7). Silica-based NPs can be utilized as efficient theranostic agents for simultaneous drug delivery and US imaging [583–584]. MSNs are inorganic materials that do not respond to temperature changes. Therefore, temperature-sensitive polymers can be used to provide temperature-responsive drug release properties to MSN–polymer hybrids. These components act as gatekeepers which can be opened by increasing the temperature during ultrasonication. Hyperthermia can induce the breakage of linkages between MSNs and capping molecules [542,585]. Similarly, US-sensitive polymers can also act as gatekeepers [20,586]. Ultrasound irradiation can change the hydrophobicity of US-sensitive polymers, thus altering the conformation and leading to the opening of the pores and cargo release [20]. Anirudhan et al. prepared thermosensitive MSNs that were grafted with tetrahydropyranyl methacrylate-*co*-aminoethyl methacrylate. Ultrasound irradiation caused bond cleavage and hydrolysis of the tetrahydropyran leading to cargo release. This polymer acted as a gatekeeper which was responsive to both temperature and US [585]. Similarly, Li et al. fabricated sodium alginate–CaCl_2_ cross-linked MSNs as an US-responsive cargo carrier. The coated polymer acted as an US-responsive gatekeeper which underwent reversible responses to both low- and high-intensity US. Ultrasound irradiation disrupted the interaction between Ca^2+^ and sodium alginate, while Ca^2+^ ions present in the physiological environment reformed the cross-links after cessation of stimuli. The bond cleavage was attributed to cavitation processes and not thermal effects. The best release behavior was observed with pulsed US treatment [587]. In another study, this group of researchers described a core–shell MSN-based structure for drug delivery, which was both pH and acoustic responsive. Moreover, US irradiation resulted in cargo release due to cavitation [588].

Lv et al. combined MSNs with MBs for US-triggered tumor therapy and contrast enhancement for US imaging. They functionalized the particles with folate for targeting purposes. Microbubble destruction via cavitation was the most probable mechanism of drug release and contrast enhancement [589]. Phospholipid-stabilized hydrophobic MSNs could promote cavitation at their surfaces and MB formation under US exposure, which led to contrast enhancement. When the lipids were in the gel phase below their melting temperature, p@hMSN generated detectable MBs after exposure to US, indicating that the lipid was effective for MB generation and contrast enhancement [590]. Paris et al. designed MSNs that displayed submicrometer-sized cavitation nuclei and evaluated their extravasation and biodistribution using US-induced inertial cavitation. The cavitation nuclei increased the efficacy of extravasation and decreased the pressure needed by 50% in comparison with MSNs alone [591]. MSNs can act as chemo-sonodynamic agents. The gas which is filled inside the cavities acts as a nucleus for cavitation under US exposure [592]. Ho et al. fabricated multifunctional superhydrophobic MSNs loaded with perfluorodecyltriethoxysilane plus DOX and β-cyclodextrin as a pore cap to prevent unwanted drug release. Under US irradiation, interfacial nanobubbles were produced on the superhydrophobic surface due to cavitation, which resulted in disruption of the tumor blood vessels. This led to enhanced penetration of the drug and reduced the tumor perfusion and eventually damaged the tumor. Moreover, this structure allowed sonodynamic therapy and caused ROS generation after ultrasonication. Ultrasound irradiation also triggered drug release. This structure could be tracked via real-time US imaging thanks to its contrast enhancement properties. The structure was well-dispersed in water and showed stability and slow biodegradation [593].

Xu et al. loaded macrophages with hollow MSNs loaded with DOX and PFP to make the so-called “cell bombs”. In this case, it was possible to use macrophages that migrated to the tumor location as targeted cargo delivery systems. Under short-pulsed HIFU sonication, MBs were formed which destroyed the nanostructures and the cells at the same time leading to drug release [594].

Ligand-conjugated MSNs can be used as contrast agents for targeted US imaging [595]. Di Paola et al. reported a biocompatible method for in vitro molecular imaging of hepatocellular carcinoma cells via targeting the glypican 3 protein (GPC3). A novel GPC3-targeting peptide was conjugated to fluorescent silica nanoparticles to enhance ultrasound contrast [596]. Qi et al. conjugated a cell-penetrating peptide to MSNs which were loaded with the Wnt3a protein to increase mesenchymal stem cell survival and enable simultaneous US imaging [597]. Kempen et al. fabricated a dual functional MSN to enhance US/MRI signals and increase cell survival through a sustained release of insulin-like growth factors inside mesenchymal stem cells [598].

Silica nanostructures can also be used in US-based therapies, cargo delivery, and imaging. Chen et al. fabricated exosome-like silica NPs through emulsion templating from the silica precursors bis(triethoxysilyl)ethane (BTSE) or bis(3-trimethoxysilylpropyl)amine (TSPA). The TSPA structures showed 40% of exosome-like morphology and allowed for US contrast enhancement. These components also produced a positively charged structure (zeta potential) for labeling negatively charged cells and for improving cell uptake. The ELSs (exosome-like silica NPs) showed the strongest echogenicity compared to other NPs with similar mass concentrations leading to a reduced dosage and better biocompatibility. Enhanced contrast was due to the discoid shape and curvature of the particles which led to more effective US backscattering at the interface. They found that the US signal increased as the size of the particles increased from 125 to 160 nm. These particles could also act as cargo carriers [599]. Gao et al. developed a hybrid between silica NPs and polyelectrolyte microcapsules which was sensitive to US irradiation. SiO_2_ was formed during the precipitation process inside or on the polyelectrolyte shell after hydrolysis of tetraethyl orthosilicate. The silica composite capsules were broken into small fragments after US exposure. The quantitative measurements of cargo release showed 30%, 66%, and 80% of release after 2, 6, and 120 s of irradiation, respectively. For high-power US, it only took a few seconds for capsule breakage, whereas under lower US power they survived longer. The morphology and mechanical properties of the capsules could be adjusted by varying the temperature, time, and amount of precursor components [28].

Fe–SiO_2_ nanoshell hybrids can be used in the color Doppler ultrasound technique [600]. The same researchers described gas-filled iron–silica nanoshells as an US contrast agent [601]. Shevchenko et al. demonstrated that dextran-coated silica NPs plus US irradiation displayed synergistic antibacterial effects. It was reported that silica NPs and dextran-coated silica NPs showed 35% and 72% reduction in bacterial viability, respectively. The higher activity of dSiNPs might be due to the dextran layer allowing better adhesion to the bacteria. Moreover, the antibacterial effect was due to bacterial membrane perforation, which allowed the contents to leak out [602]. Dextran-coated SiNPs have also been used as theranostic agents and sonodynamic therapy agents for cancer cells [603].

#### Fuel-free synthetic micro-/nanomotors

Recently, micromotors and nanomotors that can function within biological environments have attracted much attention. One important question faced during the design of these motors is where should the energy required to power them come from? Many synthetic micromotors have been designed to run on noxious fuels, so it may be difficult to utilize them in vivo unless the fuel and catalyst materials are replaced by biocompatible substitutes [604–605]. Some efforts have been made in order to design tiny motors which can run on biologically compatible fuels, such as glucose or urea. However, fuel concentration higher than physiological levels are required and low velocity hinders further progress in this field. Different approaches, including the use of electroactive polymers to encapsulate higher amounts of the enzyme [606] or creation of hollow mesoporous structures to increase the reactive surface area [607] were applied to increase the speed [608], directionality [609], and to boost enzymatic motor activity. The physiological conditions of the targeted tissue can also stimulate micro-*/*nanomotors to become active. The micro-*/*nanomotors comprised of materials such as zinc, manganese, or calcium carbonates, which decompose in a slightly acidic environment, can be activated in environments like the stomach or in the vicinity of cancer cells [610–611]. External magnetic fields can also be used as a propulsion source to direct the synthetic micro-*/*nanomotors towards the area of interest. Ultrasound and light can also be utilized to power and control synthetic micromotors. These micromotors can be functionalized with biomolecules or chemical compounds in order to carry out similar activities than catalytic enzymes [612]. Some micromotors can be powered by US for drug, gene, or protein delivery. The advantages are high-loading efficiency, lower toxicity, and more convenient control to enhance the delivery of therapeutic agents to treat diseases such as cancer [613]. These motors are physically driven micro-/nanomotors which are activated without any fuel by an external power source such as US [614]. The delivery of DOX into cancer cells has been carried out with three-segment nanowire motors. These nanomotors allowed cargo release by using a pH-sensitive polymeric segment, a magnetic-responsive function provided by a nickel segment, and a third additional gold segment for functionalization [615]. Ultrasound was used as the power source and a magnetic field was used to control the localization. Drug release was achieved by the photothermal effects produced after external NIR light delivery which was absorbed by the Au segment [616].

The group of Wang also described nanowire motors that were modified with a pH-responsive polymer and powered by US for caspase-3 delivery [617]. These polymer-modified nanomotors protected caspase-3 from release until they were taken up by the cells after US irradiation, and then the pH-responsive polymer coating was dissolved in high lysosomal pH, resulting in caspase-3 release and cell apoptosis. This delivery strategy could be applied to a variety of therapeutic proteins [613].

A new category of biological hybrid nanomotors has been recently developed with biocompatibility, biodegradability, and ability to interact with body tissues [618]. Biological organisms can be designed to contain the cargo as well as the micro-*/*nanomotor [619] due to their high physiological adaptability [620] and their ability to avoid the immune system [621]. These systems display bioavailability and a high cell affinity [622] and can enhance drug uptake by using cells or microorganisms as drug carriers [623]. Cell-based structures, such as red blood cells, bacteria, or stem cells can effectively act as cargo carriers [624]. These carriers are biocompatible and their membranes protect the cargo from rapid clearance by phagocytosis and hepatic metabolism and limit the unnecessary interaction of drugs, leading to a decreased drug toxicity and enhanced intracellular delivery due to their efficient endosomal escape mechanisms [625]. In this sense, Wu et al. designed Fe_3_O_4_ NP-loaded red blood cells (RBCs) or “red blood cell motors”, benefiting from US for propulsion [626]. The RBCs contained quantum dots as imaging agents, DOX as an anticancer drug, and magnetic NPs to enable magnetic guidance [627]. Encapsulation of DOX inside RBCs led to three times lower toxicity than free DOX over a 24 h incubation period. White blood cells can also be used to transfer cargo. They offer exceptional targeting ability as they can independently recognize tumor cells or pathogens and eradicate those via phagocytosis [628].

#### Other ultrasound-responsive nanostructures

Danti et al. reported the use of boron nitride nanotubes (BNNTs) plus synergistic US irradiation for the stimulation of human osteoblasts. BNNTs are ceramic nanomaterials with unique chemical and physical properties due to the polar nature of the B–N bonds. BNNT shows piezoelectric properties in which mechanical stress is converted into electric energy. Ultrasound irradiation can remotely activate BNNTs and cause the production of an electrical current in order to stimulate cells and tissues. The BNNTs were coated with poly(ʟ-lysine) (PLL) to enhance particle solubility. The interaction between the negatively charged cell membrane and the positive charge of PLL played an important role in cellular uptake. The human osteoblasts were incubated with BNNTs and exposed to US to convert mechanical energy into electrical stimulation. As a result, the treated osteoblasts showed significant mineralization, increased synthesis of TGFβ, and osteogenic differentiation [629].

Li et al. demonstrated that piezoelectric materials could act as sonosensitizer agents through an interesting mechanism. They tested black phosphorous nanosheets plus US irradiation to render the black phosphorous nanosheets polarized, resulting in electron bandgap widening and ROS production. Superoxide was produced from oxygen at the conduction band and hydroxyl radicals from water at the valance band [630].

Sonication can trigger the autophagy process within cells and can affect cell survival and death. Jawaid et al. investigated the synergistic effect of platinum NPs plus US irradiation in cell death. Platinum nanoparticles used alone could suppress the induction of apoptosis and autophagy due to their ROS scavenging property; however, when combined with US the opposite effect was observed with increased cell death and evidence of pyknosis. They concluded that platinum NPs could enhance the cytotoxic effects of US, and conversely, US could suppress the protective effects of platinum NPs [155].

Jin et al. proposed a novel concept for the US-induced release of nitric oxide for safe and effective gas therapy under MRI guidance. Nitric oxide is an endogenous free radical molecule with many therapeutic effects. It plays a role in the signaling process leading to vasodilation and is also involved in the modulation of the innate immune system. They fabricated BNN6-SPION@hMSN (SPION: superparamagnetic iron oxide NPs, hMSNs: hollow mesoporous silica nanoparticles) in which the BNN6 compartment released NO under US irradiation, the SPIONs acted as an MRI contrast agent, and hMSNs acted as a drug carrier [148].

Cu-based nanostructures have been used as sonosensitizer agents. Wang et al. demonstrated the sonosensitizing activity of copper–cysteamine (Cu–Cy) NPs. They stated that the synergistic combination of Cu–Cy plus US irradiation led to higher ROS generation compared to Cu–Cy or US alone. The Cu–Cy NPs could also be used in photodynamic therapy, activated by different wavelengths of the electromagnetic spectrum [631]. The presence of glutathione in cells scavenges the ROS produced via sonodynamic therapy and reduces its efficacy. Zhong et al. fabricated multifunctional PtCu_3_ nanocages with a cubic structure and a size in the range of 11–17 nm as sonosensitizing agents. They reported three different mechanisms for this preparation. First, the PtCu_3_ nanocages were able to generate ROS under US irradiation. Moreover, this nanostructure exhibited enzyme-like activity which led to two other functions. PtCu_3_ nanocages converted H_2_O_2_ molecules into OH free radicals under acidic conditions. These nanostructures could also mimic glutathione peroxidase and oxidize glutathione reducing its antioxidant activity, which led to more efficient sonodynamic therapy. They also suggested that the PtCu_3_ nanocages could be used in dual-modality imaging. Photoacoustic imaging was enabled by their high absorption of NIR light, and computed imaging was enabled by their high attenuation of X-rays. These PtCu_3_ nanocages also showed minimal toxicity for normal tissue. PtCu_3_ nanocages have been fabricated via a solvothermal method and then PEGylated with poly(maleic anhydride-alt-1-octadecene)-polyethylene glycol. In this study, 1,3-diphenylisobenzofuran was used as a trapping agent for singlet oxygen (^1^O_2_), *o*-phenylenediamine as a trapping agent for OH free radicals, and non-fluorescent 2',7'-dichlorodihydrofluorescein diacetate as a probe to measure intracellular ROS levels [632]. Similarly, Liang et al. fabricated Pt–CuS Janus nanoparticles loaded with the sonosensitizer tetra-(4-aminophenyl) porphyrin in order to enhance the efficacy of sonodynamic therapy. This structure was also capable of being used for photothermal therapy, PA imaging, and NIR thermal imaging. The Pt atoms in this structure acted as a nanoenzyme which converted H_2_O_2_ to O_2_ and also enhanced the photothermal performance. This structure was coated with a thermosensitive polymer which could regulate the enzymatic activity of Pt and release the TAPP in response to temperature changes [633]. In another study, Sun et al. fabricated Pd@Pt nanoplates with the organic sonosensitizer meso-tetra(4-carboxyphenyl)porphine (T790) in order to treat multidrug-resistant bacterial infection via sonodynamic therapy. Pd@Pt nanoplates acted as nanoenzymes with peroxidase, oxidase, and catalase-like activities and enhanced the efficacy of sonodynamic therapy. They reported that modification of Pd@Pt nanoplates with the T790 sonosensitizer decreased the inherent enzymatic activity; however, this was recovered under US irradiation. This US-switchable structure exhibited good stability, effective accumulation, reduced unwanted toxicity, and enhanced therapeutic efficacy due to its controllable nature. This structure could also be used in multimodal imaging (PA, CT, and MRI) [634].

A nickel ferrite/carbon nanocomposite (NiFe_2_O_4_/C) was fabricated by Gorgizadeh et al. and tested as a sonosensitizer agent for melanoma. This structure also exhibited MR imaging contrast enhancement and magnetic-induced hyperthermia. The US pulse profile and particle concentration affected the efficacy. The NiFe_2_O_4_/C nanocomposite not only caused ROS generation under US irradiation but also mediated the cavitation process. They stated that the synergistic cytotoxic effects of NiFe_2_O_4_/C nanocomposite plus US irradiation were due to hyperthermia and ROS generation [635]. The same researchers in a similar study fabricated MnFe_2_O_4_ and a carbon nanocomposite (MnFe_2_O_4_/C) for photothermal therapy, sonodynamic therapy, and MR imaging contrast enhancement for melanoma. They reported only a slight decrease in cell viability using the particles alone, or with NIR light exposure alone, or under US irradiation alone. However, the simultaneous combination of NIR light and US led to significant cytotoxicity and tumor necrosis [636].

Li et al. prepared a novel biomimetic nanostructure complex based on the membrane of red blood cells, which was loaded with Ag_2_S quantum dots as a sonosensitizer and the natural antitumor drug phenethyl isothiocyanate to increase H_2_O_2_ concentration and enhance the efficacy of sonodynamic therapy. This structure also had fluorescent imaging capability. Ag_2_S quantum dots were synthesized by heating up to 150 °C a solution of diethyldithiocarbamic acid silver salt, octadecene, and dodecanethiol until complete removal of water, followed by the addition of n-hexane. In the next step, Ag_2_S quantum dots were incorporated in Pluronic F-127 micelles to prevent the adsorption and aggregation of proteins in the biological microenvironment. The Ag_2_S quantum dot–Pluronic particles were then coated with RBC membranes. The RBC membranes improved the EPR effect, providing longer blood circulation, low immunogenicity, biocompatibility, and low cytotoxicity. It was shown that this structure was able to catalyze the decomposition of H_2_O_2_ to produce O_2_ and enhance the effect of sonodynamic therapy. The rate of O_2_ generation was concentration-dependent. They proposed that the mechanism of ROS generation involved sonoluminescence. In this phenomenon, the quantum dots could be excited by visible light emitted via the collapse of ultrasonic cavitation bubbles. They hypothesized that Ag_2_S quantum dots could also be excited by US irradiation as well as visible light. They showed that the rate of ROS production was higher than that of TiO_2_ NPs [637].

Dong et al. used CaCO_3_ hollow NPs as a template in order to synthesize nanosized pH-responsive sonosensitizer agents for enhanced sonodynamic therapy. They incorporated meso-tetra-(4-carboxyphenyl)porphine as the sonosensitizer (which could be activated by low-frequency US) which was bridged via its carboxyl groups to ferric irons as a metallic center, and ʟ-buthionine sulfoximine as an inhibitor of glutathione biosynthesis. This multifunctional hybrid produced ROS under US irradiation, which led to Ca^2+^ overload and mitochondrial dysfunction, while BSO inhibited the production of glutathione which intensified the ROS damage. These three mechanisms together caused extensive tumor damage and cell death [638].

Bernard et al. synthesized AgCu NPs modified with either phenanthroline or polyvinyl alcohol and tested the synergistic effects with 1MHz US irradiation on the viability of A2780 human ovarian carcinoma cells. They showed that the AgCu NPs modified with phenanthroline reduced the cell viability more than the same NPs modified with polyvinyl alcohol. They showed that US irradiation did not change the properties of the NPs. Both NPs with different surface coatings reduced cell viability in synergism with US. They hypothesized that the probable mechanism was sonoporation and increased cell membrane permeability as a result of ARF or MB oscillations. Moreover, the aggregation of the particles as a result of US was another possibility [639].

### Clinical trials on ultrasound-responsive nanomaterials

Nanotechnology has offered exceptional opportunities for therapeutic and diagnostic purposes. In vitro and in vivo studies are necessary at the primary stages since biological organisms are generally accepted as highly complex systems with many parameters in which minor alterations, such as administration of nanomedicines, can result in some unpredicted outcomes. However, these studies do not demonstrate the safety and efficacy of the use of nanomedicines in humans; therefore, clinical trials are required. The number of clinical trials on nanomedicine has been increasing in recent years, and most of them have investigated nanomedicine formulations. Few clinical trials are investigating US-responsive nanomaterials. According to clinicaltrials.gov, MB and thermosensitive liposomes are the only US-responsive nanomaterials under investigation in clinical trials, and no other nanomaterials were found in this data depository (Table 1). Some of these clinical trials are still in progress, but the outcome of some completed clinical trials are discussed here. The efficient use of US-responsive MBs in cancer treatment, image-guided therapy, and contrast enhancement has been demonstrated in completed clinical trials with published data.

**Table 1:** Clinical trials on ultrasound-responsive nanomaterials (clinicaltrials.gov).

Nanomaterial	NCT number	Application	Status

SonoVue + recombinant tissue plasminogen activator	NCT01678495	sonothrombolysis	completed
microbubble	NCT00671411	enhanced ultrasonography of blood flow in kidney masses	completed
microbubble	NCT02321527	enhanced ultrasonography	completed
SonoVue	NCT00829413	enhanced ultrasonography	completed
SonoVue + FOLFIRINOX	NCT04146441	enhanced chemotherapy	recruiting
SonoVue	NCT03477019	breast and colorectal cancer treatment	recruiting
microbubble	NCT04431674	breast cancer treatment	recruiting
microbubble	NCT04431648	head and neck cancer treatment	recruiting
microbubble	NCT04290767	enhanced ultrasonography in patients with shock	recruiting
microbubble	NCT03458975	targeted chemotherapy	recruiting
lyso-thermosensitive liposomal (LTSL) doxorubicin	NCT02181075	targeted chemotherapy of liver tumors	completed
lyso-thermosensitive liposomal (LTSL) doxorubicin	NCT02536183	pediatric cancer treatment	recruiting
lyso-thermosensitive liposomal (LTSL) doxorubicin + cyclophosphamide	NCT03749850	primary breast tumor treatment	recruiting

Dimcevski et al. [640] have investigated the safety and potential toxicity of gemcitabine in addition to US-responsive microbubbles with concurrent image guidance in inoperable pancreatic cancer patients. The primary goal of this study was to investigate the effect of sonoporation on the efficacy of chemotherapy. This combined treatment did not induce any additional toxicity or increased the frequency of side effects compared to gemcitabine chemotherapy alone. Moreover, the treatment increased patient tolerance to the increased number of gemcitabine cycles, decreased the maximum tumor diameter (in five patients), and enhanced the median survival of patients from 8.9 to 17.6 months. Through a different cancer treatment approach, a study with 28 participants demonstrated the feasibility, safety, and efficacy of combining US-triggered MB destruction with hepatocellular carcinoma radioembolization [641]. There was a greater prevalence of tumor response in the patients who received this treatment while their liver function was preserved.

Multiple clinical trials have assessed the efficacy and safety of HIFU with SonoVue, a phospholipid-shelled ultrasound contrast agent containing sulfur hexafluoride, for the treatment of uterine fibroids. SonoVue can lower the US ablation energy, shortening the sonication time to reach massive grayscale change. No serious adverse events were found, and it has been revealed that SonoVue would enhance the ablative effects of HIFU treatment in uterine fibroids. It has been suggested that cavitation is an enhancing factor in this process [642–646]. In a similar clinical trial with 102 participants, the efficacy and safety of SonoVue in the treatment of adenomyosis were investigated. SonoVue could safely enhance the HIFU ablation and early massive grayscale change, lower total energy, and reduced mean power were suggested as potential safety factors [647].

A study on lyso-thermosensitive liposomal doxorubicin (NCT02181075) is the sole completed clinical trial on the US-responsive liposomes with published data [648]. In this study, ten patients received a single intravenous infusion (50 mg/m^2^) of lyso-thermosensitive liposomal doxorubicin (LTLD), followed by extracorporeal focused ultrasound exposure of a single target liver tumor. This treatment led to an average increase of 3.7 times in doxorubicin concentration in the tumor site. Neutropenia and anemia were the adverse effects in some patients, and no death occurred. This study demonstrated the safety and feasibility of enhanced intratumoral drug delivery and chemo-ablative treatment in human liver tumors refractory to standard chemotherapy.

Although various kinds of US-responsive nanomaterials have been invented, only MBs and liposomes have entered the clinical trials. The relative biocompatibility and low toxicity of MBs and liposomes might be the pivotal reason for this. On the other hand, the uncertainty about the fate of other nanomaterials in biological systems and their potential toxicity may have hindered their progress to the clinical trials. This issue should be addressed considering that other nanomaterials, including non-organic and polymeric nanomaterials, offer many other mechanisms of action, making the treatment and diagnosis process more efficient and accurate. The possible solution to this problem might be to investigate the fate of nanomaterials in biological systems and increase their biocompatibility.

## Conclusion

In recent years, there has been a trend towards more specific, accurate, and efficient medical procedures using innovative nanomaterials. Smart drug delivery systems as a novel approach use nanostructures and their exceptional properties to gain more control over the process of delivery. Stimuli-responsive nanostructures are among the most efficient tools to be used in these systems. In this paper, the synergistic biomedical applications of nanomaterials combined with US irradiation have been comprehensively reviewed. Ultrasound devices are recognized as noninvasive for diagnosis and imaging and can also be used in drug delivery using certain stimulus-responsive nanomaterials. These US-responsive nanomaterials provide better imaging quality and on-demand drug delivery. Microbubbles, nanoemulsions, polymeric structures, lipid vesicles, surfactant-based micelles, and inorganic nanoparticles including gold NPs, titania nanostructures, carbon nanostructures, and silica nanostructures have been explored as US-responsive materials. Likewise, the mechanisms of action are also rather diverse and include cavitation, hyperthermia, ADV, ARF, and free radical and ROS generation. These mechanisms explain the US responsiveness of nanomaterials and allow for the rational design of complex hybrid composite structures. The synergistic effects of the combinations can, in many cases, be pronounced since these nanomaterials tend to have only low intrinsic toxicity and the US parameters which are employed are insufficient to damage the cells or tissues to any significant extent. Cavitation, hyperthermia, and ADV can all lead to bubble formation which can enhance the contrast of imaging modalities. Cavitation and hyperthermia can induce tissue damage, thermal lesions, and even tissue ablation. Free radical and ROS generation is the basis of sonodynamic therapy, which is another important mechanism of action of US-responsive materials. Particles can penetrate into or accumulate within the desired location, which can be explained by the ARF phenomenon. This also facilitates drug delivery and cargo release in the intended tissue. The delivery of drugs or biological cargos inside the cells may be due to sonoporation, or to the creation of transient repairable pores in the cell membrane. Cargo release from the nanomaterials can also be triggered by the degradation or disruption of the nanostructures as a result of the US energy absorbed. In summary, these novel US-responsive nanomaterials can provide more accurate and specific medical procedures with a high degree of temporal and spatial control.

The variety of US-responsive nanomaterials gives us the opportunity to select the most appropriate material for an intended application. The selection of a suitable nanomaterial, considering the pathophysiology of the disease that should be treated or diagnosed, is the first step towards creating an efficient US-responsive theranostic system. Each US-responsive nanomaterial is tied to at least one mechanism of action which determines the consequences of the administration of that specific nanomaterial in certain applications. The hybridization of different US-responsive nanomaterials with each other leads to hybrids with multiple arms to do desired functions. The required mechanism of action is an essential factor in the selection of a suitable nanomaterial. Toxicity, biocompatibility, biodegradability, and bioavailability should also be taken into account when choosing the most suitable nanomaterial; however, the route of administration and the nature of application would determine the minimum requirements in this sense. For instance, toxicity, biocompatibility, and biodegradability are more significant factors to be considered in parenteral delivery when compared with transdermal delivery. Hybridization and functionalization are further steps to optimize the treatment and diagnosis processes.

In spite of various innovations in this field, the biocompatibility and safety of these systems are still questionable, and there is a need for more studies, especially in animals, over a long term. Moreover, organ-specific accumulation and targeting ability of these nanostructures are not entirely understood, and more accurate studies are required to guarantee minimal side effects. We suggest that combining these materials with biotechnology approaches might help to overcome some of these barriers and limitations.

On the other hand, many US-responsive nanomaterials are designed for therapeutic and diagnostic purposes, and their efficacy is shown through in vitro and in vivo studies. However, few of them have undergone clinical trials and the variety of tested nanomaterials and targeted diseases investigated in clinical trials is limited. In contrast with in vitro and in vivo studies, clinical trials require higher safety and the lack of well-developed nanomaterials in terms of safety might be the reason why clinical trials are limited on many of these newly invented nanomaterials. Moreover, nanotechnology and materials scientists tend to create more novel structures with exceptional properties rather than make an effort to optimize previously created nanomaterials and translate them into the clinic. More cooperation between these scientists, biologists, and clinicians may help the society to overcome this hurdle.

Besides the therapeutic possibilities that US-responsive nanomaterials offer, the role of these materials in imaging is still rudimentary. Many imaging modalities are now available to detect and localize biological structures and processes. Some of them produce an image of structural features, while others produce images of functional processes. Combining several different imaging modalities together could create an integrated multimodality imaging device to provide higher quality diagnostic capabilities. Since nanomaterials can be fabricated in various forms and as hybrids, it will be possible to create nanostructures that are responsive to several different triggers or stimuli both internal and external. One attractive approach is to combine the benefits of the deep tissue penetration of external US irradiation with an additional responsiveness to internal physiological stimuli, such as enzyme activity, redox potential, pH changes, or increased temperature, all of which can be characteristic of specific disease states. In this case, the responsiveness of the nanomaterials to the tissue microenvironment can lead to alterations in the response of the probe to US irradiation and provide additional information about the functional properties of the tissue or organs.
